# Galactic outflows and star-formation quenching: a JWST perspective

**DOI:** 10.1007/s10509-026-04655-4

**Published:** 2026-10-20

**Authors:** Rebecca L. Davies, Sirio Belli

**Affiliations:** 1 Centre for Astrophysics and Supercomputing, Swinburne University of Technology, John Street, Hawthorn, 3122 VIC Australia; 2 Dipartimento di Fisica e Astronomia, Università di Bologna, Bologna, Italy

**Keywords:** Galaxy evolution, Galactic winds, Galaxy quenching, Post-starburst galaxies, AGN host galaxies

## Abstract

Outflows driven by active galactic nuclei (AGN) are widely considered a key mechanism for regulating and quenching star formation in massive galaxies. However, direct observational evidence linking outflows to quenching has remained elusive for decades. This article reviews recent findings for massive galaxies at cosmic noon (z∼2), based on sensitive James Webb Space Telescope (JWST) observations tracing both star formation histories (through Balmer absorption lines) and neutral outflows (through blueshifted Na i d absorption). Neutral outflows are found in $\sim 25\text{ -- }50\%$ of massive ($\log (M_{*}/M_{\odot })> 10$) galaxies, independent of star formation rate. Outflows from star-forming galaxies are consistent with being powered by star-formation and/or AGN activity, whereas outflows from quiescent systems are generally attributed to current or previous AGN activity. There is growing evidence that recent rapid quenching of star-formation is associated with powerful AGN-driven outflows with mass loading factors $\eta \gtrsim 1\text{ -- }10$. The neutral gas dominates the outflow mass budget but in most cases its velocity is below the halo escape velocity, indicating that this gas may form part of a galactic fountain. Detections of neutral gas inflows and residual cold gas reservoirs in quiescent galaxies suggest that other feedback mechanisms such as AGN heating are required to keep galaxies quenched over long timescales. We highlight the potential of multiphase outflow measurements to constrain feedback models, describe the main systematic uncertainties in mass outflow rate measurements, and discuss how the z∼2 results inform our understanding of the formation of the earliest massive quiescent galaxies.

## Introduction

Understanding what causes massive galaxies to quench is one of the biggest outstanding problems in modern astrophysics (see Bluck 2026a,b; Whitaker and Bezanson 2026 for reviews). Cosmological hydrodynamical simulations (e.g. Illustris-TNG; Pillepich et al. 2018, EAGLE; Schaye et al. 2015, SIMBA; Davé et al. 2019, Magneticum; Steinborn et al. 2015) generally require some form of feedback from active galactic nuclei (AGN) to reproduce the observed galaxy stellar mass function, star-formation rate distribution and color bimodality (see also Benson Astron et al. 2003; Di Matteo et al. 2005; Springel and Hernquist 2005; Bower et al. 2006; Croton et al. 2006; Hopkins et al. 2006; Somerville et al. 2008; Erb 2015; Beckmann et al. 2017). However, the exact physical processes coupling AGN accretion energy to the host galaxy remain a matter of debate. Feedback processes are often divided into two broad categories: ejective or quasar-mode feedback, which expels gas from galaxies through outflows, and preventative or radio-mode feedback, which heats gas, preventing it from cooling and being re-accreted to replenish the reservoir of fuel for star-formation (see Harrison and Ramos Almeida 2024 and references therein). The state-of-the-art simulations mentioned above employ vastly differing combinations of ejective and preventative feedback and yet, because of the large number of free parameters that can be tuned, they produce galaxies with roughly the same stellar properties (e.g. Crain and van de Voort 2023; Wright et al. 2024).

Observational studies have taken a variety of approaches to constrain the primary driver(s) of quenching. For example, large statistical studies of galaxies spanning $z\sim 0\text{ -- }2.5$ have investigated which properties most strongly predict whether a galaxy is quenched. While stellar mass is a good predictor (Peng et al. 2010), central density, velocity dispersion, or bulge mass are even better predictors (e.g. Franx et al. 2008; Lang et al. 2014; Bluck et al. 2022). This result is often interpreted as a sign that long-term AGN heating of halo gas may be the primary quenching mechanism. However, directly constraining the timescale and causes of quenching is substantially more challenging. Most local quiescent galaxies quenched $\sim 8\text{ -- }10\text{ Gyr}$ ago (e.g. Ilbert et al. 2013), but spectroscopic signatures of rapid quenching and the powerful outflows that may trigger it disappear after $\sim 1\text{ Gyr}$ (e.g. Park et al. 2024; Sun et al. 2024; Taylor et al. 2026). Therefore, to robustly identify the physical processes that quench massive galaxies, it is necessary to study galaxies that are currently undergoing, or recently underwent, quenching.

To this end, studies of post-starburst galaxies have provided important insights into galaxy quenching mechanisms. Post-starburst galaxies are distinguished by weak ionized gas emission lines and strong Balmer absorption lines, indicating a dominant population of A-stars (Dressler and Gunn 1983). The prevalence of A stars combined with the absence of O and B stars suggests that these galaxies must have experienced a strong burst of star-formation a few hundred Myr prior to the epoch of observation, followed by rapid quenching.

Post-starburst galaxies are rare at z∼0, accounting for <1% of all massive galaxies (e.g. Zabludoff et al. 1996; Quintero et al. 2004; Goto 2007; Alatalo et al. 2016; Chen et al. 2019). Prior to JWST, there were very few high-quality Balmer absorption line spectra for galaxies beyond the local Universe, driving the development of alternate methods to identify post-starburst galaxies, primarily using combinations of rest-frame colors (e.g. Wild et al. 2014; Belli et al. 2019). Number densities calculated using these methods suggest post-starburst galaxies are more common at z∼2, accounting for $\sim 5\%$ of all massive galaxies (e.g. Wild et al. 2016). Furthermore, as many as half of all galaxies entering the red sequence at z∼2 could go through the post-starburst phase (e.g. Forrest et al. 2018; Rowlands et al. 2018; Belli et al. 2019; D’Eugenio et al. 2020; Wild et al. 2020; French 2021; Skarbinski et al. 2026). The other half likely quench over longer timescales ($\sim 1\text{ Gyr}$) comparable to the typical gas depletion times at these redshifts (e.g. Tacconi et al. 2020). This slow quenching is thought to be consistent with starvation, i.e. the cessation of gas replenishment due to inflows (e.g., Peng et al. 2015).

High-velocity outflows are found in approximately half of all massive post-starburst galaxies at z∼0 (e.g. Davis et al. 2012; Alatalo et al. 2016; Baron et al. 2020, 2022), and these outflows appear to decline in strength and velocity with time since quenching (e.g. Sun et al. 2024), suggesting that the sudden quenching is driven by rapid ejection of cold gas. However, prior to JWST, there were very few direct constraints on the mass outflow rates from post-starburst galaxies outside of the local Universe. Early studies of post-starburst galaxies at $0.5 \lesssim z \lesssim 2$ were based on small samples and/or stacked spectra and revealed tentative evidence for widespread, high velocity outflows similar to what is observed at z∼0 (e.g. Tremonti et al. 2007; Maltby et al. 2019; Man et al. 2021; Taylor et al. 2024). Deep ALMA observations of post-starburst galaxies at local and intermediate redshift (z≲0.6) have revealed that their molecular gas reservoirs also decline rapidly with time after quenching, at a rate faster than can be explained by the residual star-formation activity in these systems, providing further evidence for significant ejection of molecular gas by outflows (e.g. French et al. 2018; Bezanson et al. 2022; Baron et al. 2023). Measuring molecular gas reservoirs in more distant post-starburst galaxies is very challenging even with sensitive sub-mm telescopes like ALMA and NOEMA. Initial observational results based on small samples support the z∼0 picture that gas reservoirs decline rapidly with time after quenching, but there is substantial diversity in the amount of molecular gas measured for different samples (e.g. Williams et al. 2021; Belli et al. 2021; Whitaker et al. 2021; Zanella et al. 2023; Suess et al. 2025). Although there is compelling circumstantial evidence for a link between outflows and rapid quenching, robust measurements of outflow rates and molecular gas reservoirs for post-starburst galaxies at cosmic noon are needed to verify this directly.

The unprecedented near-infrared sensitivity of JWST has transformed our understanding of AGN-driven outflows and their link with quenching in massive galaxies at cosmic noon. This review outlines those advances, with a specific focus on insights gained from the first observations of neutral outflows in representative samples of galaxies beyond the local Universe.

## Situation prior to JWST

### Multiphase gas in galaxies and outflows

The interstellar medium (ISM) is multiphase, containing cold molecular gas ($T\sim 10\text{ -- }100\text{ K}$, $n\sim 10^{3-6}\text{ cm}^{-3}$), cool neutral atomic gas ($\sim 100\text{ K}$, $\sim 30\text{ cm}^{-3}$), warm neutral and ionized gas ($\sim 10^{4}\text{ K}$, $\sim 0.1\text{ -- }1\text{ cm}^{-3}$), and hot diffuse gas (∼10$^{5-6}\text{ K}$, $\sim 10^{-3}\text{ cm}^{-3}$) (e.g. Field et al. 1969; McKee and Ostriker 1977; Wolfire et al. 1995; Cox 2005; Saintonge and Catinella 2022). The hot and warm ionized phases have low densities but large filling factors, and in the Milky Way these phases account for $\sim 20\text{ -- }25\%$ of the total ISM mass (e.g. Ferrière 2001; Girichidis et al. 2020). The remaining 75% of the ISM mass is found in the denser neutral and molecular phases (Draine 2011; Saintonge and Catinella 2022). The neutral phase is dominant at z∼0 (e.g. Boselli et al. 2014; Catinella et al. 2018), but the molecular phase is expected to account for a larger fraction of the mass at higher redshifts due to the conversion of H i to H_2_ at high gas mass surface densities (e.g. Tacconi et al. 2020).

Outflows comprise a combination of hot wind (∼10$^{6}\text{ K}$) material (e.g. supernova ejecta or material launched from black hole accretion disks) and gas swept-up from the ISM (e.g. King and Pounds 2015; Bieri et al. 2017; Richings and Faucher-Giguère 2018; Fielding and Bryan 2022). Cool gas may also precipitate out of the hot wind due to radiative cooling in turbulent mixing layers (e.g. Gronke and Oh 2018; Ji et al. 2019; Fielding et al. 2020; Chen et al. 2023). As a result, outflows consist of multi-phase gas spanning many orders of magnitude in temperature and density (e.g. Rupke et al. 2005b; Bolatto et al. 2013; Cicone et al. 2014; Feruglio et al. 2015; Veilleux et al. 2020). Disk-scale ISM simulations of both supernova-driven winds (e.g. CGOLS; Schneider et al. 2018, TIGRESS; Kim et al. 2020, QUOKKA; Vijayan et al. 2024) and AGN-driven outflows (e.g. Ward et al. 2024) predict that similar to the ISM, the majority of the mass ejected in outflows is found in the cooler neutral and molecular phases. Consistent with this prediction, multiphase studies of outflows in the local Universe generally find that ionized gas outflow rates are comparable to or lower than atomic and/or molecular mass outflow rates, although there is significant variation in measured ratios between studies (e.g. Fiore et al. 2017; Oosterloo et al. 2017; Fluetsch et al. 2019; Avery et al. 2022; Mazzilli Ciraulo et al. 2025; Hernández-Yévenes et al. 2026).

### Outflows at cosmic noon

The detection of prominent blueshifted rest-frame UV absorption lines in stacked spectra of Lyman Break Galaxies provided one of the earliest indications that outflows with velocities of several hundred km s^−1^ are widespread in star-forming galaxies at cosmic noon (e.g. Pettini et al. 2001; Adelberger et al. 2003; Shapley et al. 2003; Steidel et al. 2010). The next key breakthroughs came from near-infrared integral field spectroscopy studies which enabled detailed measurements of outflow properties. Observations of luminous AGN (quasars, bright radio galaxies, and AGN-hosting ultraluminous infrared galaxies) revealed galaxy-scale outflows extending 5 – 10 kpc from the nuclei. These outflows were estimated to release a comparable amount of energy over their lifetimes to the gravitational binding energy of their host galaxies, establishing AGN-driven outflows as a potentially important mechanism for quenching star-formation at cosmic noon (e.g. Nesvadba et al. 2008; Alexander et al. 2010; Harrison et al. 2012; Brusa et al. 2015; Zakamska et al. 2016). Some works reported evidence for localised suppression of star-formation in the paths of powerful outflows (e.g. Cano-Díaz et al. 2012; Cresci et al. 2015; Carniani et al. 2016), although subsequent studies have shown that these conclusions are very sensitive to assumptions regarding the dominant ionization mechanism and the spatial distribution of dust across the systems (e.g. Scholtz et al. 2021; Kakkad et al. 2023).

A key question raised by these studies was whether powerful outflows are only driven by the most luminous AGN. Over the past decade, near-infrared galaxy surveys have revealed that >50% of massive ($\log (M_{*}/M_{ \odot})> 11$) galaxies at cosmic noon show ionized outflows whose properties are most consistent with being AGN-driven (see Förster Schreiber and Wuyts 2020, and references therein). These outflows have typical velocities of 1000 – 2000 km s^−1^, exceeding the halo escape velocities and enabling long-term removal of star-forming gas (e.g. Förster Schreiber et al. 2014; Genzel et al. 2014; Harrison et al. 2016; Leung et al. 2019; Förster Schreiber et al. 2019). Most ionized outflow emission originates from the central 2 – 3 kpc (e.g. Förster Schreiber et al. 2014; Genzel et al. 2014), although fainter high velocity emission is detected to $\gtrsim 5\text{ kpc}$ (e.g. Davies et al. 2020); consistent with the outflow extents measured for higher luminosity AGN (e.g. Harrison et al. 2012; Cresci et al. 2015). These high-velocity nuclear outflows have distinctly different properties from star-formation driven winds, which are most prevalent in galaxies above the main sequence (e.g. Förster Schreiber et al. 2019) and originate from kiloparsec-scale star-forming clumps that are often offset from galaxy nuclei (e.g. Genzel et al. 2011; Newman et al. 2012b). Star-formation driven outflows have typical velocities of 300 – 600 km s^−1^; below the escape velocities of their host galaxies, suggesting that most material launched in star-formation-driven outflows will eventually return to the galaxies in galactic fountains (e.g. Davies et al. 2019; Förster Schreiber et al. 2019; Freeman et al. 2019). Resolved observations suggest that outflows from individual star-forming clumps may propagate $\sim 1\text{ kpc}$ from their launch point (e.g. Newman et al. 2012b), with most star-formation-driven outflow emission confined within the effective radius of the host galaxies (e.g. Newman et al. 2012a).

Despite the high incidence of fast outflows in typical massive galaxies at z∼2, the mass outflow rates measured from ionized gas emission line tracers are generally lower than the star-formation rates of the host galaxies, in contrast with findings for more luminous AGN (e.g. Fiore et al. 2017) and predictions from galaxy formation models (e.g., Nelson et al. 2019; Mitchell et al. 2020). These results suggest that although outflows are a prominent feature of typical massive galaxies at cosmic noon, they may not have a major impact on the cycle of gas and are therefore unlikely to be a major cause of quenching (e.g. Förster Schreiber et al. 2019; Concas et al. 2022). However, as discussed in Sect. 2.1, ionized gas likely traces only a small fraction of the outflowing material. Thus, measurements of the cool gas mass carried by distant outflows are crucial to understand their impact on molecular gas reservoirs and their role in galaxy quenching.

Low-ionization absorption lines in rest-frame UV spectra (e.g. Mg ii, Fe ii, Si ii, O i) have ionization potentials close to hydrogen and thus trace neutral or partially ionized gas. Although blueshifted rest-frame UV absorption lines have been detected in many star-forming galaxies at cosmic noon (e.g. Weiner et al. 2009; Steidel et al. 2010; Kornei et al. 2012; Erb 2015), very few studies report mass outflow rates measured from these lines because 1) the fraction of the total outflowing mass probed by any tracer of partially ionized gas has a large associated uncertainty (e.g. Chisholm et al. 2016) and 2) the optical depth is degenerate with the small-scale covering fraction of gas against the background continuum source. This degeneracy can be broken using doublet features such as Mg ii for which the equivalent width ratio of the lines varies with optical depth. However, Mg ii absorption is often saturated, providing only a lower limit on the outflowing mass. Another key limitation of rest-frame UV absorption line tracers is that they require a bright rest-frame UV continuum, and thus do not provide a viable means of probing outflows in very dusty galaxies or quenching systems (e.g. Kehoe et al. 2024).

Most measurements of molecular outflows at cosmic noon prior to JWST came from sub-millimeter observations of CO emission lines in quasars (e.g. Carniani et al. 2017; Vayner et al. 2017; Brusa et al. 2018; Chartas et al. 2020; Vayner et al. 2021; Zhong et al. 2023), although some possible detections have also been reported in main sequence galaxies (e.g. Herrera-Camus et al. 2019). Deep stacking analyses using large samples of main sequence galaxies have yielded non-detections of CO outflows down to stringent limits (e.g. Barfety et al. 2025; Langan et al. 2026). These CO outflow non-detections do not preclude the presence of a dominant molecular gas component in outflows from typical star-forming galaxies; this gas may be heated by AGN ionizing radiation, suppressing the CO emission and leading to brighter emission in warm molecular gas tracers such as mid-infrared $H_{2}$ lines (e.g. Davies et al. 2024a; Fernández-Ontiveros et al. 2026).

**Fig. 1:**
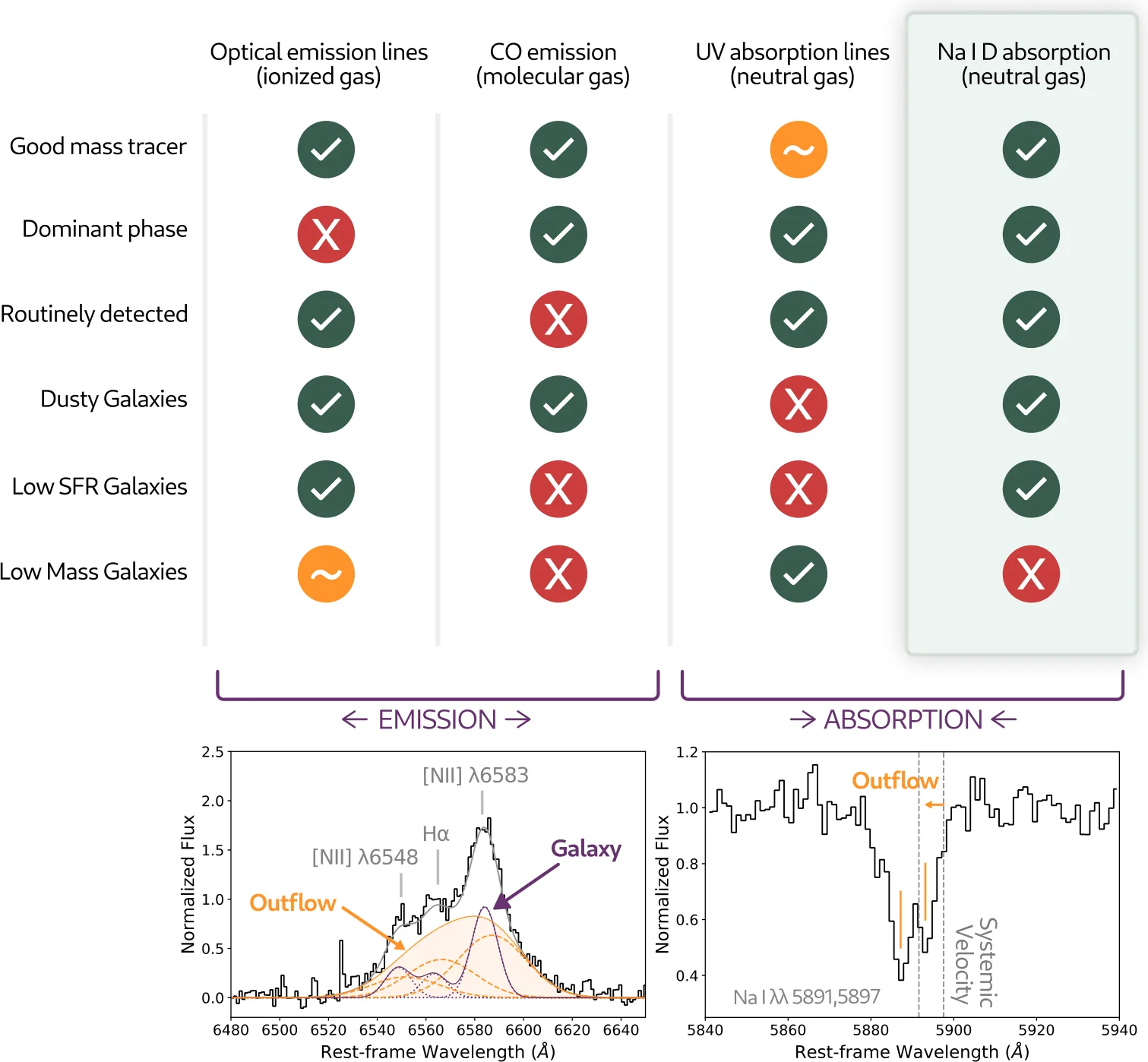
Strengths and weaknesses of outflow tracers commonly used at cosmic noon (top). The differences between emission line and absorption line tracers are illustrated using zoom-ins of an example z∼2 galaxy spectrum (bottom; Davies et al., in prep). Broad optical emission line components tracing ionized outflows are detected in many galaxies at cosmic noon, but they do not trace the dominant outflow phase and often are not sensitive to outflows in low mass galaxies because they blend with the much stronger ISM emission. CO outflows are normally only detected in the brightest systems. UV absorption lines require a bright rest-frame UV continuum and generally cannot be used to probe outflows from dusty or quenching galaxies. Na i d absorption is the only tracer that is routinely detected even in dusty and low SFR galaxies and is a good tracer of the mass in the dominant outflow phase. However, it is generally not detected in low mass galaxies, likely due to insufficient dust shielding. Bottom left: Emission-line outflows manifest as broad components (orange) super-imposed on narrower emission from the host galaxy (purple). Dotted and dashed curves show Gaussian profiles of individual emission lines, and solid curves show the summed profile of each component. Bottom right: Absorption-line outflows manifest as blueshifted components relative to the galaxy systemic velocity

Figure 1 summarizes the advantages and disadvantages of the main observational tracers used to study outflows from massive galaxies at cosmic noon.

### Na I D as a neutral outflow tracer

In the local Universe, a common tracer of neutral outflows is Na i d, i.e., the Na I $\lambda \lambda $5891, 5897 doublet absorption (e.g. Heckman et al. 2000; Chen et al. 2010; Concas et al. 2019; Roberts-Borsani and Saintonge 2019; Avery et al. 2022; Weng et al. 2026). Neutral sodium has an ionization potential of 5.1 eV – significantly lower than hydrogen – and therefore exists primarily in neutral regions where it is shielded by significant columns of gas and dust (e.g. Savage and Sembach 1996; Baron et al. 2020). Na i d absorption is prominent in the spectra of late-type stars and therefore the stellar contribution must be fitted and removed prior to modelling the interstellar contribution. The sodium doublet is resonant, meaning that the interstellar component can appear in either emission or absorption. In the local Universe, galaxies with stellar masses below $\log (M_{*}/M_{\odot})\simeq 10$ typically show Na i d in emission. Massive, low SFR galaxies show weak absorption at the galaxy systemic velocity, which is typically attributed to excess stellar absorption. The most massive, highly star-forming galaxies show blueshifted Na i d absorption, tracing neutral outflows (e.g. Chen et al. 2010; Concas et al. 2019; Roberts-Borsani and Saintonge 2019; Avery et al. 2022).

Na i d mitigates many of the issues associated with other outflow tracers at cosmic noon (Fig. 1). Unlike rest-frame UV lines, Na i d absorption is routinely detected in dusty and low SFR galaxies, and it is typically not saturated, providing robust constraints on optical depth and covering fraction (Chen et al. 2010; Weng et al. 2026). The main limitation of Na i d absorption is that it is typically undetected in low mass galaxies, likely due to insufficient dust shielding in lower metallicity environments (e.g. Roberts-Borsani and Saintonge 2019). Unfortunately, at z≳0.7, Na i d is shifted to the near-infrared, where contamination from the Earth’s atmosphere makes it very difficult for ground-based telescopes to reach the sensitivity required to robustly detect stellar continuum emission at high signal-to-noise ratio.

## Advances from JWST: neutral outflows and their connection to rapid quenching

**Fig. 2:**
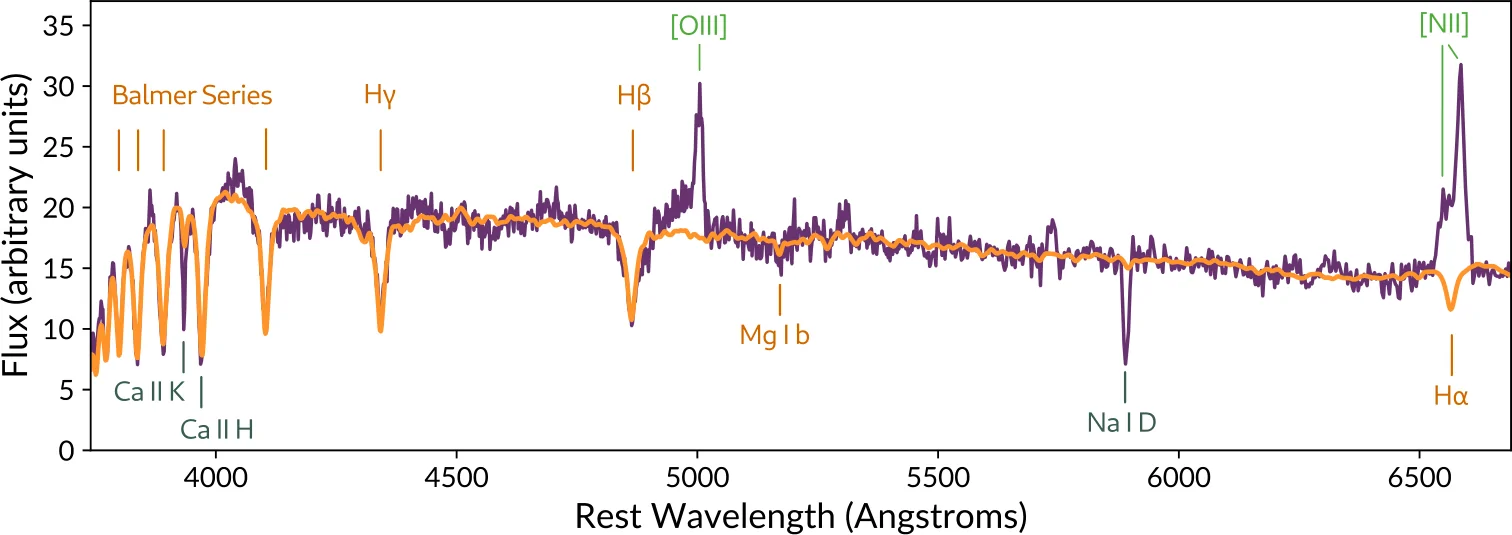
JWST/NIRSpec medium-resolution ($\mathrm{R} \simeq 1000$) spectrum of galaxy COSMOS-11142 at z=2.445 from GO 1810 (Belli et al. 2025). The data is shown in purple and the best-fit Prospector stellar continuum model is shown in orange. Key emission lines are marked in light green, stellar absorption lines are marked in orange, and interstellar absorption lines tracing neutral outflows are marked in dark green

The unprecedented near-infrared sensitivity and wavelength coverage of JWST has driven major advances in our understanding of the role of outflows in quenching massive galaxies at cosmic noon. The quality of the data now available is illustrated in Fig. 2, which shows a deep JWST/NIRSpec spectrum of a massive galaxy at z∼2.4 observed using the G140M, G235M and G395M filters with integration times of 13 h, 3.2 h, and 1.6 h, respectively. The galaxy exhibits weak emission lines and strong Balmer absorption lines, characteristic of a post-starburst system. Furthermore, the presence of deep, blueshifted Na i d absorption reveals a powerful neutral outflow. Thanks to JWST, we now have access to high signal-to-noise ratio spectra over the entire rest-frame optical wavelength range for hundreds of massive galaxies at cosmic noon, making it possible to measure the mass of neutral gas ejected by outflows, investigate their link to AGN activity and examine the impact of these outflows on galaxy star-formation histories.

The vast majority of Na i d outflow studies at cosmic noon to date have been conducted using JWST/NIRSpec medium resolution spectra similar to what is shown in Fig. 2. These gratings provide a spectral resolution $R = c / \Delta v \simeq 1000$, corresponding to a velocity full width at half maximum of $\sim 300\text{ km}\,\mathrm{s}^{-1}$. These observations can robustly identify outflows with bulk line-of-sight blueshifts $\gtrsim 100\text{ km}\,\mathrm{s}^{-1}$, which translates to outflow velocities $\gtrsim 200\text{ km}\, \mathrm{s}^{-1}$(e.g. Davies et al. 2024b; Sun et al. 2026a).

In this section, we summarize recent findings on the demographics and properties of neutral outflows in massive z∼2 galaxies (Sect. 3.1), their driving mechanisms (Sect. 3.2), the role of outflows in quenching star-formation (Sect. 3.3), and the long-term evolution of massive quiescent galaxies at cosmic noon (Sect. 3.4).

### Outflow demographics

The spectrum shown in Fig. 2 was obtained as part of the Blue Jay survey, a JWST Cycle 1 program (GO 1810) which targeted a mass-selected sample of 150 galaxies at 1.7<z<3.5 with the goal of examining the causes of galaxy quenching (Belli et al. 2025). Here, we highlight results from the Blue Jay survey which was the first program to comprehensively examine the properties of neutral outflows at z∼2, but we note that the key findings have since been independently confirmed by several other JWST surveys, including EXCELS, DeepDive, and JADES (Kehoe et al. 2026; Sun et al. 2026a; Taylor et al. 2026; Zhu et al. 2026).

**Fig. 3:**
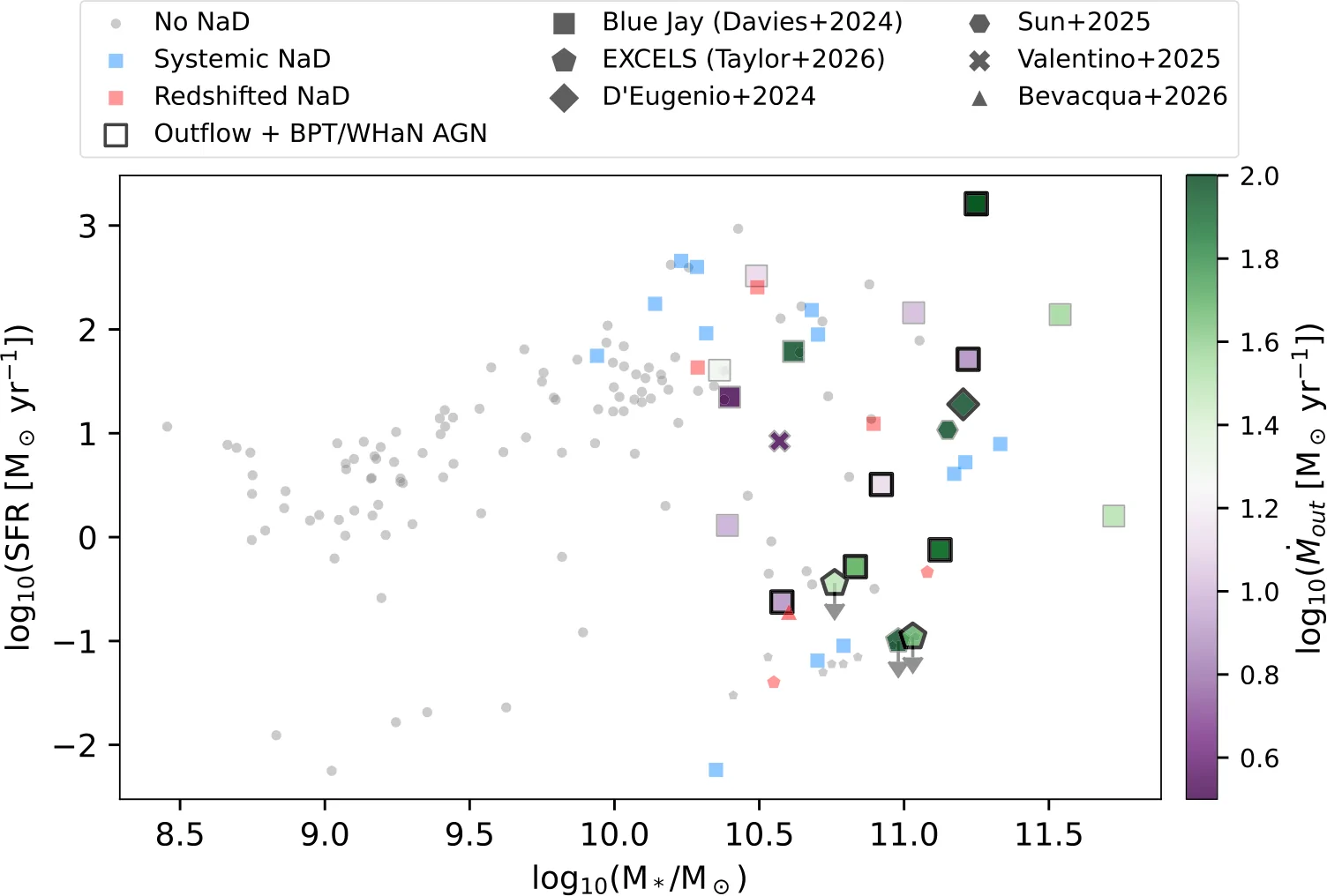
Demographics of neutral outflows at 1.3<z<4.6 detected via Na i d absorption. Grey dots are galaxies from the Blue Jay sample with no Na i d absorption. Small blue and red markers indicate detections of systemic and redshifted Na i d absorption, respectively, from both Blue Jay and other samples (differentiated by the marker shape). Large green-to-purple markers indicate galaxies with Na i d outflows, color-coded by mass outflow rate. Black outlines denote outflow galaxies classified as AGN either using both the BPT and WHaN diagrams when [O iii] and Hβ are robustly measured, or using the WHaN diagram alone

Strong Na i d absorption tracing neutral gas was detected in $\sim 50\%$ of massive galaxies ($\log (M_{*}/M_{\odot})>10$) from the Blue Jay sample, spanning a range of star-formation rates from star-forming to quiescent systems (Fig. 3; Davies et al. 2024b). Notably, the Na i d absorption detected in z∼2 quiescent galaxies is an order of magnitude stronger than what is seen in quenched systems at z∼0 and cannot be explained by excess absorption in stellar atmospheres, even when allowing for elevated sodium abundances. Na i d absorption is very rarely detected in lower mass galaxies, consistent with z∼0 results. Stacking analysis demonstrates that this is not due to weaker continuum flux in low mass galaxies (e.g. Davies et al. 2024b) and may instead reflect the requirement for significant dust shielding to prevent sodium from being ionized (e.g. Roberts-Borsani and Saintonge 2019).

Within the Blue Jay sample, half of the Na i d absorption detections are blueshifted by at least 100 km s^−1^ relative to the galaxy systemic velocity, indicative of neutral outflows. A further 39% are consistent with the galaxy systemic velocity and may trace outflows with lower (projected) velocities, or gas in the ISM. The existence of neutral outflows is further confirmed through the detection of blueshifted absorption in the Ca ii H and K lines, which are also resonant and thus trace cool neutral gas (Liboni et al. 2026). The inferred neutral gas outflow rates ($\dot{M}_{\mathrm{neutral}}$) are at least as large as the galaxy SFRs, corresponding to mass loading factors $\eta = \dot{M}_{\mathrm{neutral}}/\mathrm{SFR} \gtrsim 1$, and the outflows have velocities of 200 – 1000 km s^−1^, lower than or comparable to the galaxy escape velocities (Davies et al. 2024b).

When both neutral (Na i d absorption) and ionized gas (optical emission line) outflows are detected, the neutral phase dominates the outflow mass budget but travels more slowly than the ionized gas (e.g. Belli et al. 2024; D’Eugenio et al. 2024; Bugiani et al. 2025; Wu 2025), consistent with the canonical picture of multiphase winds developed from detailed studies of nearby outflows (e.g. Leroy et al. 2015). Overall, these results suggest that the majority of massive galaxies at cosmic noon host powerful neutral outflows that rapidly eject large amounts of cool gas from galaxies but likely do not permanently remove this material from the host halos.

### Outflow driving mechanisms

In galaxies with little current star-formation, several lines of evidence suggest that the observed outflows are primarily driven by processes linked to supermassive black hole accretion such as radiation pressure, accretion disk winds, or radio jets. Many quiescent galaxies show high-ionization emission lines characteristic of radiative AGN activity (e.g. elevated [O iii]/Hβ or [Ne iii]/[O ii] ratios or [Ne v]λ3427 emission; Davies et al. 2024b; Belli et al. 2024; D’Eugenio et al. 2024; Bugiani et al. 2025; Wu 2025; Sun et al. 2026a; Taylor et al. 2026; Valentino et al. 2026). In other cases, the spectra are dominated by strong low-ionization emission lines (e.g. elevated [N ii]/Hα and [S ii]/Hα ratios; Bugiani et al. 2025; Sun et al. 2026a; Taylor et al. 2026) which could trace either low luminosity AGN or shock excitation by radio jets (e.g. Davis et al. 2012; Ilha et al. 2022; Couto and Storchi-Bergmann 2023). We note that >50% of all [N ii]/Hα-selected AGN at cosmic noon do not show AGN signatures at X-ray or radio wavelengths, likely because the available observations are not sensitive to moderate luminosity AGN at these distances (e.g. Förster Schreiber et al. 2019). Ionized outflows from galaxies with low levels of star-formation often reach velocities of 800 – 1000 km s^−1^ (e.g. Belli et al. 2024; D’Eugenio et al. 2024; Bugiani et al. 2025), consistent with typical AGN-driven outflows and substantially higher than typical star-formation-driven outflows at z∼2 (see discussion in Sect. 2.2). In most cases, the rates of energy and/or momentum injection by supernovae are insufficient to power the observed outflows, whereas the detected AGN are able to supply the required energy (e.g. Davies et al. 2024b; Sun et al. 2026a).

It is important to consider whether the observed Na i d absorption in quiescent galaxies could trace ‘fossil’ outflows, meaning outflows that were launched by past star-formation or AGN activity that has since faded. This is especially relevant for post-starburst galaxies which have experienced a recent episode of strong star formation that could be responsible for the observed outflow. However, it is unclear how long outflows remain observable once the driving source fades. Previous studies suggest that starburst-driven outflows can persist for a maximum of 25 Myr (McQuinn et al. 2018), significantly shorter than the typical post-burst timescales which are hundreds of Myr. AGN flicker on much shorter (Myr) timescales, and hydrodynamical simulations of outflows in idealized galaxy bulges predict that in gas-poor galaxies, fossil outflows should outnumber outflows powered by current AGN activity (Zubovas and Maskeliūnas 2023). Consistent with this picture, there are multiple examples of quiescent galaxies that show powerful outflows but little or no evidence for optical (radiatively efficient) AGN activity, potentially revealing the signatures of past AGN episodes (e.g. Almaini et al. 2025; Sun et al. 2026b,a; Taylor et al. 2026). It is also possible, however, that these outflows are driven by AGN while in a low-luminosity (or radiatively inefficient) state (e.g. Bugiani et al. 2025; Hewitt et al. 2026). A key unknown in determining whether fossil outflow scenarios are plausible is how long ago the outflows were launched. To calculate this, we need to know how far the outflows have traveled from their point of launch. This is very poorly constrained, with current estimates ranging from 1 kpc to 40 kpc (see Sect. 5). A 200 km s^−1^ outflow 1 kpc from the galaxy would have a travel time of 5 Myr, meaning that the current SFR should be similar to the SFR when the outflow was launched. The same outflow at a distance of 40 kpc would have a travel time of $\sim 200\text{ Myr}$; comparable to observed post-burst timescales and making a fossil star-formation-driven outflow scenario more plausible.

For main sequence galaxies, it is more difficult to conclusively determine the outflow driving mechanism. Initial indications suggest that galaxies with Na i d-detected outflows have a higher AGN incidence than the underlying massive galaxy population. However, the ongoing star-formation activity provides sufficient energy and momentum to power the observed outflows, and there is tentative evidence that the outflow velocity and mass outflow rate are weakly correlated with the level of star-formation activity (e.g. Davies et al. 2024b; Sun et al. 2026a). Measuring the ionized outflow velocities in these systems would help to distinguish between AGN-driven and SF-driven winds, which have typical ionized outflow velocities of 1000 – 2000 km s^−1^ and 500 km s^−1^, respectively (e.g. Förster Schreiber et al. 2019).

### Role of outflows in quenching massive galaxies

Neutral outflows are observed in 25 – 50% of galaxies with $\log (M_{*}/M_{\odot})> 10$ at cosmic noon (see Sect. 3.1). To understand how these outflows impact galaxy evolution and star-formation quenching, it is important to examine their link with star-formation histories. There are now several known examples of massive, quenched $z\sim 1\text{ -- }4$ galaxies that experienced a rapid decline in star-formation activity within the last 300 Myr – 1 Gyr and show powerful neutral outflows with $\eta \gtrsim 1\text{ -- }10$, sufficient to quench star-formation (e.g. Belli et al. 2024; D’Eugenio et al. 2024; Wu 2025; Sun et al. 2026b). These case studies provide the most direct observational evidence thus far that gas removal by powerful AGN-driven ejective feedback drives rapid star-formation quenching in massive galaxies at cosmic noon.

Statistical studies of quiescent galaxies suggest a tentative link between post-quenching timescale and outflow properties. Within the z∼2 Blue Jay sample, neutral outflows are detected in 75% of galaxies that quenched within the last 500 Myr, whereas the neutral outflow fraction is 0% for more evolved systems (Park et al. 2024). Similarly, all ionized outflow detections are in galaxies $< 500\text{ Myr}$ post-quenching (Bugiani et al. 2025). In the slightly higher redshift EXCELS and *DeepDive* samples ($z\sim 3 \text{ -- }4$), 23 – 30% of galaxies $\leq 500\text{ Myr}$ post-quenching show neutral outflows (Taylor et al. 2026; Zhu et al. 2026), whereas older galaxies at the same redshift do not show neutral outflows (e.g. Forrest et al. 2026). In z∼1 post-starburst galaxies, Mg ii outflows are detected up to 1 Gyr after the last burst (Taylor et al. 2024). These findings are broadly consistent with studies of starburst, post-starburst and quiescent galaxies at z∼0 which have shown that the incidence and velocity of Na i d outflows decline as galaxies age and quench (e.g. Sun et al. 2024). Park et al. (2024) found that the oldest quenched galaxies at z∼2 show weak absorption consistent with the galaxy systemic velocity, perhaps tracing older, lower-velocity outflows that are undetectable in the $\mathrm{R} \sim 1000$ JWST observations.

These observational results establish powerful outflows as a compelling candidate to explain rapid quenching of massive galaxies at cosmic noon. Previous studies have found evidence for two quenching pathways, defined by their characteristic timescales: fast quenching ($\sim 100\ \mathrm{--}\ 300\text{ Myr}$) and slow quenching ($\sim 1\text{ Gyr}$) (e.g. Wu et al. 2018; Belli et al. 2019; Carnall et al. 2019; Suess et al. 2021; Park et al. 2023). Slow quenching is often attributed to starvation of gas supply and depletion of remaining gas by star-formation (e.g. Larson et al. 1980; Peng et al. 2015; Spitoni et al. 2017; Trussler et al. 2020). On the other hand, fast quenching is hypothesised to be triggered by dramatic events like mergers or violent disk instabilities which funnel gas towards the centres of galaxies, trigger centrally concentrated starbursts and AGN accretion, and drive powerful outflows (e.g. Hopkins et al. 2006; Barro et al. 2013; Dekel and Burkert 2014; Zolotov et al. 2015; Tacchella et al. 2016). There is some evidence that rapid star-formation quenching at cosmic noon is preceded by bursts of star-formation (e.g. Park et al. 2023; Belli et al. 2024; D’Eugenio et al. 2024; Sun et al. 2026b). However, only the strongest bursts can be recovered from the observed spectroscopy in a way that is robust to the choice of star-formation history prior (e.g. Park et al. 2024). If outflow-driven quenching is triggered by galaxy compaction events, then we would also expect the strongest outflows to be found in starburst galaxies, as is the case in the local Universe (e.g. Sun et al. 2024). Starburst galaxies are rare and not well represented in current samples (e.g. Davies et al. 2024b; Sun et al. 2026a). There are tentative indications that galaxies with higher specific SFRs may drive faster and more energetic outflows (e.g. Sun et al. 2026a), but the uncertainties on individual measurements are large due to many factors including low velocity resolution, unknown outflow geometry and uncertainties in converting Na i d absorption measurements to hydrogen column densities (see Sect. 5). Observations of larger galaxy samples at higher spectral resolution are needed to determine the relationship between bursts of star-formation and strong outflows at cosmic noon.

### Long-term evolution of massive quiescent galaxies

While there is compelling evidence that powerful ejective feedback can trigger rapid quenching, it is less clear how these quenched galaxies evolve over longer timescales. Davies et al. (2024b) find that 71% of neutral outflows at z∼2 are too slow to escape the host halo, in contrast with z∼2 AGN-driven ionized outflows which typically exceed the galaxy escape velocity (e.g. Förster Schreiber et al. 2019; Leung et al. 2019; Belli et al. 2024; D’Eugenio et al. 2024; Bugiani et al. 2025). This is consistent with predictions from ISM simulations that cool gas entrained in outflows moves significantly slower than the surrounding hot wind (e.g. Gronke and Oh 2018; Kim et al. 2020; Vijayan et al. 2020; Ward et al. 2024). Given the low velocities, the outflowing material may eventually be re-accreted in a galactic fountain (see also Sun et al. 2026a; Zhu et al. 2026). Indeed, Bevacqua et al. (2026) discovered a strong Na i d-detected neutral inflow feeding a massive galaxy at z=2.7 that has nevertheless been quiescent for the past 1 Gyr, and there are several other examples of quiescent galaxies with detected Na i d inflows (e.g. Davies et al. 2024b; Sun et al. 2026a; Taylor et al. 2026). Measurements of neutral gas column density and velocity as a function of galactocentric distance along multiple sightlines for individual galaxies would provide crucial insights into the fate of outflowing neutral gas.

Furthermore, it remains unclear whether powerful outflows completely remove all the cold gas from galaxies. The weak systemic absorption detected in galaxies $> 500\text{ Myr}$ post-quenching suggests that these galaxies may either still have, or recently had, significant amounts of neutral gas in their ISM. Taylor et al. (2026) found no evidence for systemic absorption in z∼3 quenched galaxies from EXCELS; however, all of their galaxies are observed less than 500 Myr after quenching when outflows are more dominant. Scholtz et al. (2026) measured a molecular gas fraction of <1% in a z∼3 massive quiescent galaxy with a strong neutral outflow, and showed that star-formation could plausibly have consumed all of the galaxy’s gas, with outflows balancing inflows to prevent replenishment of the gas reservoir. This is only one possible evolutionary history, but demonstrates the potential power of combining star-formation history, outflow and molecular gas measurements for large samples of cosmic noon galaxies at different quenching stages.

AGN heating could be a key mechanism preventing galaxies from rejuvenating. Current estimates suggest that between 20 – 50% of passive galaxies at 1<z<5 display optical emission line properties consistent with weak radiative AGN activity or shocks produced by radio jets interacting with the ISM (e.g. Slob et al. 2024; Bugiani et al. 2025; Skarbinski et al. 2026; Stevenson et al. 2026). At z∼0, old quiescent galaxies with neutral gas inflows show an increased incidence of radio jets (e.g. Roy et al. 2021; Moghni et al. 2026). Post-starburst galaxies at cosmic noon have relatively weak and compact radio jets, whereas quiescent galaxies have higher radio luminosities and more extended morphologies, suggesting that maintenance mode feedback becomes stronger as galaxies age (e.g. Patil et al. 2026).

Another possible source of long-timescale feedback is episodic weak outflows. Quenched galaxies may go through cycles of fueling and feedback, whereby inflows fuel the nuclear region, leading to increased AGN activity and outflows which remove cold gas, causing the AGN to turn off until inflows build up enough gas to trigger another AGN episode (e.g. Almaini et al. 2025; Taylor et al. 2026). Consistent with this picture, French et al. (2025) identified four local post-starburst galaxies with significant recent brightening in radio emission, and demonstrated that this newly injected energy is sufficient to drive outflows and turbulence capable of suppressing star-formation in these systems. This cycling could continue until the inflows cease or become too weak to fuel AGN activity. Observations of more quiescent galaxies over a wider range of ages are needed to understand how long episodic AGN and outflows persist in these systems.

**Fig. 4:**
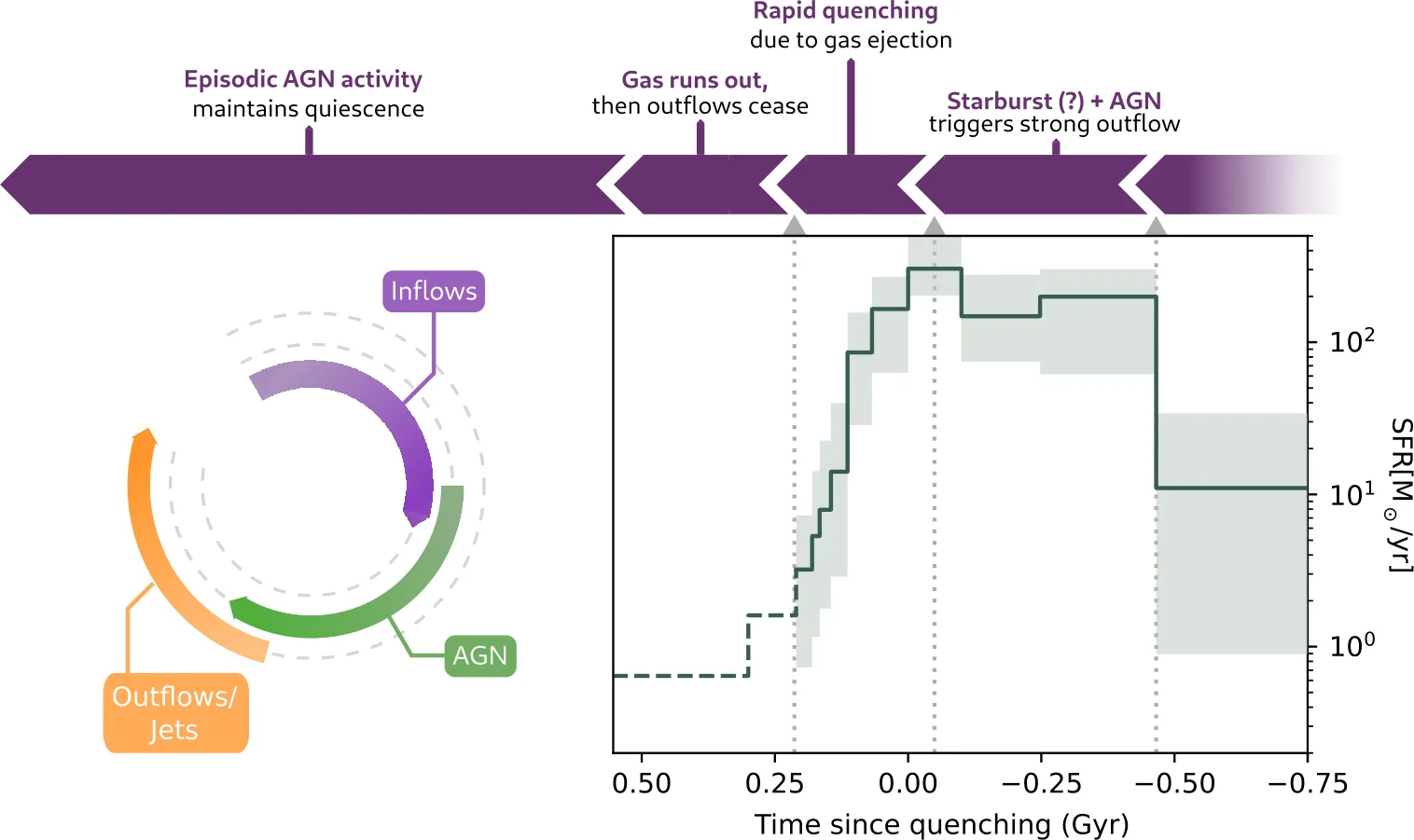
Schematic summarising the possible mechanisms driving the rapid quenching and ongoing quiescence of massive galaxies at cosmic noon. On the right, the solid green line and shaded region show the star-formation history of COSMOS-11142 (Belli et al. 2024), and the dashed line shows a possible extrapolation into the future for illustration purposes. Strong gas inflow/accretion likely triggered a burst of star-formation and AGN activity, driving a powerful outflow. This outflow ejected a large amount of cold gas, leading to rapid quenching and leaving the galaxy below the main sequence where it continues to drive a strong outflow (Belli et al. 2024). The left side of the figure illustrates what may happen to COSMOS-11142 in the future. As the gas runs out, the galaxy may enter a phase of episodic AGN activity, where gas inflows trigger AGN activity that then powers outflows or radio jets, preventing any new or remaining gas from forming stars (e.g. Park et al. 2024; Almaini et al. 2025; Taylor et al. 2026)

Figure 4 summarises the possible mechanisms driving the rapid quenching and ongoing quiescence of massive galaxies at cosmic noon.

## Testing feedback models with multiphase outflow measurements

The availability of neutral outflow measurements for large samples of massive z∼2 galaxies presents a unique opportunity to study multiphase outflow properties and test feedback models used in cosmological simulations. Feedback models are tuned to match galaxy population statistics such as the galaxy stellar mass function and the cosmic SFR density (see e.g. Somerville and Davé 2015; Naab and Ostriker 2017; Crain and van de Voort 2023 and references therein), but strong degeneracies between model parameters mean that simulations employing vastly different combinations of ejective and preventative feedback can produce galaxies with roughly the same stellar properties (e.g. Crain and van de Voort 2023; Wright et al. 2024). Directly measuring outflow properties provides a means to break this degeneracy.

One of the most fundamental observables for testing feedback models is the relationship between galaxy stellar mass ($M_{*}$) and outflow mass loading factor (η). The $M_{*}-\eta $ relation is set by a combination of stellar feedback at lower masses and AGN feedback at higher masses (e.g. Nelson et al. 2019; Mitchell et al. 2020) and thus constrains several key aspects of feedback models. In most cosmological simulations, AGN feedback becomes dominant at $\log (M_{*}/M_{\odot}) \gtrsim 10.5\text{ -- }10.7$, primarily because the feedback models are calibrated to reproduce the knee in the galaxy stellar mass function (e.g. Crain et al. 2015; Weinberger et al. 2017; Davé et al. 2019; Dolag et al. 2025). Directly measuring the mass at which the $M_{*}-\eta $ scaling changes would reveal whether the knee in the galaxy stellar mass function is indeed primarily caused by a change in the dominant feedback mode. The slope and normalization of the $M_{*}-\eta $ relation above the transition mass constrains the relative importance of ejective and preventative AGN feedback: simulations with dominant ejective feedback predict that η should increase with mass, whereas those with dominant preventative feedback predict flatter scalings and/or lower normalization (e.g. Bower et al. 2017; Zinger et al. 2020). Similarly, if low mass galaxies have significantly weaker outflows than predicted by simulations, this may indicate that the star formation is being suppressed through heating rather than direct gas ejection (e.g. Smith et al. 2024; Bennett et al. 2025).

Many observational studies have presented measurements of η as a function of $M_{*}$ (e.g. Förster Schreiber et al. 2019; Fluetsch et al. 2019; Llerena et al. 2023; Marasco et al. 2023; Carniani et al. 2024; Weldon et al. 2024). Recent compilations of multiple samples suggest that outflows from low mass galaxies are weaker than predicted by simulations, although there is significant scatter between individual measurements (e.g. Mazzilli Ciraulo et al. 2025; Davies et al. 2026; Hernández-Yévenes et al. 2026). However, the vast majority of these studies are based on ionized gas alone. Small samples of outflows measured in both ionized and cool (atomic/molecular) phases suggest that including the cool component boosts η by about an order of magnitude (e.g. Fluetsch et al. 2019; Davies et al. 2026; Hernández-Yévenes et al. 2026). Adding the cool component is also likely to change the slope of the measured $M_{*}-\eta $ relation. As discussed in Sect. 2.1, the amount of cool gas in simulated outflows depends sensitively on the balance between cloud destruction, radiative cooling, and turbulent mixing between outflow phases (e.g. Gronke and Oh 2018; Ji et al. 2019; Fielding et al. 2020; Chen et al. 2023; Ward et al. 2024). ISM simulations predict that the ionized-to-total and molecular-to-atomic ratios vary significantly as a function of star-formation rate surface density (which sets the strength of the incident radiation field; Rathjen et al. 2023) and gas density (which regulates the self-shielding in the cold gas; Vijayan and Krumholz 2024). Measuring the fraction of outflowing mass in different gas phases as a function of galaxy properties would provide valuable constraints on physical models of cold gas in galactic winds.

In summary, multiphase measurements of η for large samples of galaxies at $\log (M_{*}/M_{\odot})> 10$ would provide key constraints on the fraction of cold gas in galactic winds, the stellar mass where AGN feedback becomes dominant, and the strength of ejective feedback, especially in high mass galaxies. Measuring the cool gas outflow rates from lower mass galaxies remains challenging due to a lack of robust outflow mass tracers in these systems (see Fig. 1).

## Uncertainties on mass outflow rates

JWST has provided a wealth of insights into the properties of neutral outflows and their impact on star-formation, but outflow rate measurements are still subject to significant systematic uncertainties. Computing neutral mass outflow rates from measurements of blueshifted sodium absorption requires assumptions about 1) the sodium abundance, neutral fraction and dust depletion (to convert the gas-phase neutral sodium column density to a hydrogen column density), and 2) the geometry of the outflowing material (to convert column density to total mass and outflow rate). The Appendix outlines the key equations used in these calculations.

There are virtually no constraints on the abundance, neutral fraction or dust depletion of sodium in galactic winds, and most studies adopt canonical values measured for cold clouds in the Milky Way (Rupke et al. 2005a; Shih and Rupke 2010; Baron et al. 2022). These values may not be representative of the conditions in outflows, and there is likely to be significant object-to-object variation. Even within the Milky Way, dust depletion of Na varies by 0.5 dex (Wakker and Mathis 2000). Dust depletion generally increases in denser gas, but dust may be destroyed in outflows due to interaction with the hot wind fluid (e.g. Farber and Gronke 2022; Richie and Schneider 2026). Similarly, the neutral fraction in outflows may vary by more than an order of magnitude due to differences in radiation field strength and gas density (e.g. Murray et al. 2007; Baron et al. 2020). Constraints on sodium-to-hydrogen ratios in extragalactic environments can be obtained using absorption systems against bright background continuum sources detected in both Na i and H i. Moretti et al. (2026) detected Na i d and Lyα absorption in a Damped Lyman Alpha system at z=2.4 and measured a N(H i)/N(Na i) ratio 30% lower than the canonical value, highlighting the significant systematic uncertainties on individual outflow mass measurements. In the coming years, large surveys will significantly increase the number of extragalactic absorption systems with detections of Na i d combined with either Lyα (e.g. DESI; Weng et al. 2026; 4MOST; Peroux et al. 2023) or H i 21 cm absorption (e.g. ASKAP-FLASH; Weng et al. 2022). These statistical samples will allow us to quantify for the first time how the N(Na i)-to-N(H i) conversion factor varies with galaxy properties such as metallicity and dust attenuation. When direct N(H i) measurements are not available, measurements of other low-ionization resonant absorption lines (e.g. Ca ii, Mg ii, Fe ii) can help determine the true hydrogen column density (e.g. Valentino et al. 2025; Wu 2025; Liboni et al. 2026).

The geometry of neutral outflows is also poorly constrained. Most Na i d outflow measurements beyond the local Universe come from single aperture spectra. Resolved studies at z∼0 find that Na i d absorption is typically confined within $\lesssim 10\text{ kpc}$ of galaxy nuclei (e.g. Martin 2006; Rupke and Veilleux 2015; Rupke et al. 2017; Baron et al. 2020; Roberts-Borsani 2020; Avery et al. 2022; Rubin et al. 2022); likely due to the requirement for significant dust shielding. D’Eugenio et al. (2024) used the JWST/NIRSpec IFU to map a Na i d outflow across a z∼3 post-starburst galaxy and measured an extent of at least 2.7 kpc, consistent with the z∼0 results. Belli et al. (2024) measured outflowing ionized gas extending 3 kpc from the centre of a galaxy that also shows a strong neutral outflow. Mg ii outflows at $z\sim 0.7\text{ -- }2.0$ are similarly found to have a typical spatial extent of $\sim 5\text{ kpc}$ (Pessa et al. 2026). However, outflows are most easily mapped in face-on galaxies where their size traces the projected extent across the galaxy disk, rather than distance from the galaxy which is the key quantity for outflow rate measurements. Again, quasar absorption line measurements provide complementary constraints on outflow sizes. Moretti et al. (2026) detected neutral sodium 38 kpc from a quenched galaxy at z∼2.4 using quasar absorption line spectroscopy, suggesting that the sizes of Na i d outflows may vary by up to an order of magnitude. The results presented in Davies et al. (2024b) and Sun et al. (2026a) assume $r_{\mathrm{out}} = 1\text{ kpc}$ and thus represent a conservative lower limit on the mass outflow rates.

The solid angle Ω can be indirectly constrained through multiple channels. Using stacked spectra from the Sloan Digital Sky Survey, Chen et al. (2010) showed that face-on galaxies display more blueshifted Na i d absorption than edge-on galaxies which show primarily systemic absorption, consistent with expectations for non-spherical ($\Omega <4\pi $) outflows oriented perpendicular to the galaxy disk. The fraction of galaxies with detected outflows $f_{\mathrm{out}}$ is determined by the product of the outflow covering fraction ($C_{\Omega}=\Omega /4\pi $) and the outflow duty cycle (e.g. Xu et al. 2025). Since the duty cycle must be ≤1, this implies $C_{\Omega }\geq f_{\mathrm{out}}$. For a segment of a sphere subtending a solid angle Ω, the half-opening angle θ is given by $\Omega = 4\pi (1-\cos(\theta ))$, and thus $f_{\mathrm{cov}}=1-\cos(\theta )$. At z∼2, Na i d outflows are detected in 25 – 50% of massive galaxies (e.g. Davies et al. 2024b; Sun et al. 2026a; Taylor et al. 2026), implying wide-angle winds with half opening angles of $\theta \geq 40\text{ -- }60^{\circ}$. This is consistent with the wide opening angles measured for local starburst-driven superwinds (see Veilleux et al. 2020, and references therein) as well as outflows observed in Mg ii emission at cosmic noon (Pessa et al. 2026), and similar to or larger than opening angles measured in local Seyfert galaxies (e.g. Revalski et al. 2021). However, outflow studies using rest-frame UV absorption lines at z≳2 have reported correlations between outflow velocity and galaxy inclination, consistent with collimated outflows (e.g. Kehoe et al. 2026). Better constraints on outflow geometry will require spatially resolved mapping of Na i d outflows. Mapping Na i d outflows at lower redshifts, and especially in systems where extraplanar Na i d is detected directly in emission, will provide valuable constraints on the typical geometries of these winds.

## Summary and outlook

JWST has transformed our understanding of outflows and their role in galaxy quenching. In particular, the ability to detect neutral gas outflows and link them with galaxy star-formation histories and AGN activity has revealed the strongest observational evidence thus far for a direct link between AGN-driven ejective feedback and star-formation quenching in massive galaxies at cosmic noon.

These results may also provide important clues about how the earliest massive quiescent galaxies formed. The star-formation histories of massive quiescent galaxies at $z\sim 3 \text{ -- }4$ show prominent peaks of star-formation followed by rapid quenching (e.g., Carnall et al. 2023, 2024; Glazebrook et al. 2024; Nanayakkara et al. 2025; de Graaff et al. 2025; Edward et al. 2026), similar to what is observed at cosmic noon. Simulations predict that powerful AGN-driven outflows likely play a key role in the formation of the first massive quiescent galaxies (e.g. Kimmig et al. 2025; Lagos et al. 2025; Szpila et al. 2025; Chandro-Gómez et al. 2026). As massive quiescent galaxies are discovered at increasingly high redshift (Weibel et al. 2025), it is crucial to investigate the role of AGN-driven outflows in the rapid cessation of star formation in these systems. Observations of Na i d, Fe ii and Mg ii absorption lines have revealed the presence of neutral outflows in two quiescent galaxies at z∼4 and z∼7 (Wu 2025; Valentino et al. 2025), providing further evidence for AGN-driven ejection of gas leading to rapid quenching.

Additional insights into the formation of early massive quiescent galaxies can be gained by studying their likely progenitors. The inferred high star-formation rates prior to quenching suggest that these galaxies may be descended from z∼5 starbursts or sub-millimetre galaxies (e.g. Toft et al. 2014; Valentino et al. 2020; Araya-Araya et al. 2026). Studies based on ALMA and JWST have shown that stellar feedback in such systems can drive very powerful outflows that may exhaust their molecular gas reservoirs without the need for AGN feedback (e.g. Spilker et al. 2018, 2020; Butler et al. 2023; Davies et al. 2026). Future surveys of outflows in early massive quiescent galaxies and their progenitors would provide unprecedented insights into the physical processes leading to rapid quenching in the early Universe.

## Data Availability

The spectra in Fig. 1 will be published in an upcoming paper (Davies et al., in prep). The spectrum in Fig. 2 was published by Belli et al. (2024) and can be downloaded from the associated Zenodo repository: https://zenodo.org/records/10058978. The measurements in Fig. 3 come from a range of sources. Literature measurements are sourced directly from the relevant papers. Stellar masses, star-formation rates and star-formation histories for Blue Jay galaxies will be published in an upcoming paper.

## Appendix: Computing mass outflow rates from Na I D absorption

The first step in measuring the neutral mass outflow rate from Na i d absorption is to fit and divide out the stellar continuum, including stellar Na i d absorption, using a stellar population fitting code such as Prospector (Leja et al. 2019; Johnson et al. 2021) or ppxf (Cappellari 2023). This leaves the interstellar Na i d absorption profile as a function of velocity, $f_{\text{Na I D}}(v)$. The insterstellar Na i d profile is often parametrized using the partial covering model (Rupke et al. 2005b) which constrains the optical depth (τ) and covering fraction ($C_{f}$) of the absorbing gas against the background continuum source:

A.1 $$ f_{\text{Na I D}}(v) = 1 - C_{f} + C_{f} \exp \left (-\tau _{b}(v) - \tau _{r}(v) \right ) $$

Here, $\tau _{b}(v)$ and $\tau _{r}(v)$ are the optical depth profiles of the Na i d 5891 Å and 5897 Å lines, respectively. Most studies adopt either a Gaussian or Voigt profile for τ(v). The optical depth ratio of the line centers is fixed at $\tau _{b,0} = 2~\tau _{r,0}$, reflecting the known doublet ratio (Draine 2011). Note that this is different from the ratio of the absorption equivalent widths, which varies from 2 in the optically thin regime to 1 in the optically thick regime. The optical depth at the centre of the red line ($\tau _{r,0}$) can then be converted to Na i column density following Draine (2011):

A.2 $$ \begin{aligned} &\mathrm{N}_{\text{Na I}}\ (\mathrm{cm}^{-2}) \\ &= 10^{13} \left (\frac{\tau _{r,0}}{0.7580} \right ) \left (\frac{0.4164}{f_{\mathrm{lu}}}\right )\left ( \frac{1215\text{ \r{A}}}{\lambda _{\mathrm{lu}}}\right )\left ( \frac{b}{10~\mathrm{km}\,\mathrm{s}^{-1}}\right ) \end{aligned} $$

Here $f_{\mathrm{lu}} = 0.32$ is the oscillator strength, $\lambda _{\mathrm{lu}} = 5897$ Å is the rest-frame wavelength of the transition, and b is the Doppler parameter, related to the Gaussian velocity dispersion σ by $b = \sqrt{2} \sigma $. The Na i column density can then be converted to a total gas column density following Shih and Rupke (2010) and Baron et al. (2022):

A.3 $$ \mathrm{N}_{\mathrm{H}}\ (\mathrm{cm}^{-2}) = \frac{\mathrm{N}_{\text{Na I}}\ (\mathrm{cm}^{-2})}{(1-y) \, 10^{A+B+C}} $$

Here (1−y) is the sodium neutral fraction, A is the solar sodium abundance, B accounts for the depletion of sodium onto dust grains, and C is the metallicity relative to solar. Most studies adopt canonical values of B=−0.95 (Savage and Sembach 1996) and 1−y=0.1 (Rupke et al. 2005a) based on measurements of cool diffuse clouds in the Milky Way.

To convert the line-of-sight gas column density to a mass outflow rate, one must account for the outflow geometry. Unresolved studies of Na i d outflows typically adopt the classical ‘thin-shell’ model (e.g. Heckman et al. 2000; Martin 2005; Rupke et al. 2005b), which assumes a spherically symmetric mass-conserving shell of outflowing material at a radius $r_{\mathrm{out}}$ that covers a fraction of the solid sphere given by $C_{\Omega}$. The inferred mass outflow rate scales linearly with the size of the outflow shell which in turn scales linearly with $r_{\mathrm{out}}$ and $C_{\Omega}$. The small-scale covering fraction of the absorbing material against the background continuum source, $C_{f}$, accounts for clumpiness of the outflowing gas. Under these assumptions, the mass outflow rate is given by

A.4 $$ \begin{aligned} &\dot{M}_{\mathrm{out}}(\mathrm{M}_{\odot}\,\mathrm{yr}^{-1}) \\ &= 11.45 \, \left (C_{f} \frac{C_{\Omega}}{0.4}\right ) \left ( \frac{\mathrm{N}_{\mathrm{H}}}{10^{21}\text{ cm}^{-2}}\right ) \left(\frac{r_{\mathrm{out}}}{1\text{ kpc}}\right) \left(\frac{v_{\mathrm{out}}}{200\text{ km/s}}\right) \end{aligned} $$

The outflow size and velocity can also be combined to estimate the dynamical time $t_{\mathrm{dyn}}$:

A.5 $$ t_{\mathrm{dyn}} (\mathrm{Myr}) \simeq 5 \, \left(\frac{r_{\mathrm{out}}}{1 \text{ kpc}} \right) \left(\frac{200 \text{ km}\,\mathrm{s}^{-1}}{v_{\mathrm{out}}} \right) $$

## References

[CR1] Adelberger, K.L., Steidel, C.C., Shapley, A.E., et al.: Galaxies and intergalactic matter at Redshift : overview. Astrophys. J. **584**(1), 45–75 (2003). 10.1086/345660. arXiv:astro-ph/0210314 [astro-ph]

[CR2] Alatalo, K., Lisenfeld, U., Lanz, L., et al.: Shocked POststarburst Galaxy Survey. II. The molecular gas content and properties of a subset of SPOGs. Astrophys. J. **827**(2), 106 (2016). 10.3847/0004-637X/827/2/106. arXiv:1604.01122 [astro-ph.GA]

[CR3] Alexander, D.M., Swinbank, A.M., Smail, I., et al.: Searching for evidence of energetic feedback in distant galaxies: a galaxy wide outflow in a z ˜ 2 ultraluminous infrared galaxy. Mon. Not. R. Astron. Soc. **402**, 2211–2220 (2010). 10.1111/j.1365-2966.2009.16046.x. arXiv:0911.0014 [astro-ph.CO]

[CR4] Almaini, O., Wild, V., Maltby, D., et al.: No evidence for excess AGN activity in recently quenched massive galaxies at cosmic noon. Mon. Not. R. Astron. Soc. **539**(4), 3568–3581 (2025). 10.1093/mnras/staf659. arXiv:2504.15342 [astro-ph.GA]

[CR5] Araya-Araya, P., Cochrane, R.K., Sodré, L Jr., et al.: The connection between dusty star-forming galaxies and the first massive quenched galaxies. Astron. Astrophys. **707**, A305 (2026). 10.1051/0004-6361/202557426. arXiv:2509.26646 [astro-ph.GA]

[CR6] Avery, C.R., Wuyts, S., Förster Schreiber, N.M., et al.: Cool outflows in MaNGA: a systematic study and comparison to the warm phase. Mon. Not. R. Astron. Soc. **511**(3), 4223–4237 (2022). 10.1093/mnras/stac19010.48550/arXiv.2201.08079. arXiv:2201.08079 [astro-ph.GA]

[CR7] Barfety, C., Jolly, J.B., Förster Schreiber, N.M., et al.: PHIBSS: searching for molecular gas outflows in star-forming galaxies at . Astrophys. J. **988**(1), 55 (2025). 10.3847/1538-4357/addc6f. arXiv:2502.13226 [astro-ph.GA]

[CR8] Baron, D., Netzer, H., Davies, R.I., et al.: Multiphase outflows in post-starburst E + A galaxies - II. A direct connection between the neutral and ionized outflow phases. Mon. Not. R. Astron. Soc. **494**(4), 5396–5420 (2020). 10.1093/mnras/staa1018. arXiv:2004.04749 [astro-ph.GA]

[CR9] Baron, D., Netzer, H., Lutz, D., et al.: Multiphase outflows in post-starburst E + A galaxies - I. General sample properties and the prevalence of obscured starbursts. Mon. Not. R. Astron. Soc. **509**(3), 4457–4479 (2022). 10.1093/mnras/stab3232. arXiv:2105.08071 [astro-ph.GA]

[CR10] Baron, D., Netzer, H., French, K.D., et al.: Star formation and molecular gas properties of post-starburst galaxies. Mon. Not. R. Astron. Soc. **524**(2), 2741–2769 (2023). 10.1093/mnras/stad1792. arXiv:2204.11881 [astro-ph.GA]

[CR11] Barro, G., Faber, S.M., Pérez-González, P.G., et al.: CANDELS: the progenitors of compact quiescent galaxies at . Astrophys. J. **765**(2), 104 (2013). 10.1088/0004-637X/765/2/104. arXiv:1206.5000 [astro-ph.CO]

[CR12] Beckmann, R.S., Devriendt, J., Slyz, A., et al.: Cosmic evolution of stellar quenching by AGN feedback: clues from the Horizon-AGN simulation. Mon. Not. R. Astron. Soc. **472**, 949–965 (2017). 10.1093/mnras/stx1831. arXiv:1701.07838

[CR13] Belli, S., Newman, A.B., Ellis, R.S.: MOSFIRE spectroscopy of quiescent galaxies at . II. Star formation histories and galaxy quenching. Astrophys. J. **874**(1), 17 (2019). 10.3847/1538-4357/ab07af10.48550/arXiv.1810.00008. arXiv:1810.00008 [astro-ph.GA]

[CR14] Belli, S., Contursi, A., Genzel, R., et al.: The diverse molecular gas content of massive galaxies undergoing quenching at . Astrophys. J. Lett. **909**(1), L11 (2021). 10.3847/2041-8213/abe6a6. arXiv:2102.07881 [astro-ph.GA]

[CR15] Belli, S., Park, M., Davies, R.L., et al.: Star formation shut down by multiphase gas outflow in a galaxy at a redshift of 2.45. Nature **630**(8015), 54–58 (2024). 10.1038/s41586-024-07412-1. arXiv:2308.05795 [astro-ph.GA] PMC11153157 38648852

[CR16] Belli, S., Bugiani, L., Park, M., et al.: The Blue Jay Survey: deep JWST spectroscopy for a representative sample of galaxies at cosmic noon. arXiv e-prints (2025). 10.48550/arXiv.2510.11775. arXiv:2510.11775 [astro-ph.GA]

[CR17] Bennett, J.S., Smith, M.C., Fielding, D.B., et al.: Prevention is better than cure? Feedback from high specific energy winds in cosmological simulations with ARKENSTONE. Mon. Not. R. Astron. Soc. **543**(2), 1456–1478 (2025). 10.1093/mnras/staf1440. arXiv:2410.12909 [astro-ph.GA]

[CR18] Benson, A.J., Bower, R.G., Frenk, C.S., et al.: What shapes the luminosity function of galaxies? Astrophys. J. **599**(1), 38–49 (2003). 10.1086/379160. arXiv:astro-ph/0302450 [astro-ph]

[CR19] Bevacqua, D., Marchesini, D., Saracco, P., et al.: Feeding the dead: neutral gas inflow in a long-quenched ancient massive galaxy at observed with JWST/NIRSpec. Astrophys. J. **997**(2), 189 (2026). 10.3847/1538-4357/ae247c. arXiv:2510.11455 [astro-ph.GA]

[CR20] Bezanson, R., Spilker, J.S., Suess, K.A., et al.: Now you see it, now you don’t: star formation truncation precedes the loss of molecular gas by 100 Myr in massive poststarburst galaxies at . Astrophys. J. **925**(2), 153 (2022). 10.3847/1538-4357/ac3dfa. arXiv:2111.14877 [astro-ph.GA]

[CR21] Bieri, R., Dubois, Y., Rosdahl, J., et al.: Outflows driven by quasars in high-redshift galaxies with radiation hydrodynamics. Mon. Not. R. Astron. Soc. **464**(2), 1854–1873 (2017). 10.1093/mnras/stw2380. arXiv:1606.06281 [astro-ph.GA]

[CR22] Bluck, A.F.L.: A review of Galaxy quenching – Part I: defining the problem and observational results. arXiv e-prints (2026a). 10.48550/arXiv.2608.07221. arXiv:2608.07221 [astro-ph.GA]

[CR23] Bluck, A.F.L.: A review of Galaxy quenching – Part II: theoretical solutions and direct observational tests. arXiv e-prints (2026b). 10.48550/arXiv.2608.07247. arXiv:2608.07247 [astro-ph.GA]

[CR24] Bluck, A.F.L., Maiolino, R., Brownson, S., et al.: The quenching of galaxies, bulges, and disks since cosmic noon. A machine learning approach for identifying causality in astronomical data. Astron. Astrophys. **659**, A160 (2022). 10.1051/0004-6361/202142643. arXiv:2201.07814 [astro-ph.GA]

[CR25] Bolatto, A.D., Warren, S.R., Leroy, A.K., et al.: Suppression of star formation in the galaxy NGC 253 by a starburst-driven molecular wind. Nature **499**, 450–453 (2013) 10.1038/nature12351. arXiv:1307.6259 23887428

[CR26] Boselli, A., Cortese, L., Boquien, M., et al.: Cold gas properties of the Herschel Reference Survey. II. Molecular and total gas scaling relations. Astron. Astrophys. **564**, A66 (2014). 10.1051/0004-6361/201322312. arXiv:1401.8101 [astro-ph.GA]

[CR27] Bower, R.G., Benson Astron, J., Malbon, R., et al.: Breaking the hierarchy of galaxy formation. Mon. Not. R. Astron. Soc. **370**(2), 645–655 (2006). 10.1111/j.1365-2966.2006.10519.x. arXiv:astro-ph/0511338 [astro-ph]

[CR28] Bower, R.G., Schaye, J., Frenk, C.S., et al.: The dark nemesis of galaxy formation: why hot haloes trigger black hole growth and bring star formation to an end. Mon. Not. R. Astron. Soc. **465**(1), 32–44 (2017). 10.1093/mnras/stw2735. arXiv:1607.07445 [astro-ph.GA]

[CR29] Brusa, M., Bongiorno, A., Cresci, G., et al.: X-shooter reveals powerful outflows in X-ray selected obscured quasi-stellar objects. Mon. Not. R. Astron. Soc. **446**(3), 2394–2417 (2015). 10.1093/mnras/stu2117. arXiv:1409.1615 [astro-ph.GA]

[CR30] Brusa, M., Cresci, G., Daddi, E., et al.: Molecular outflow and feedback in the obscured quasar XID2028 revealed by ALMA. Astron. Astrophys. **612**, A29 (2018). 10.1051/0004-6361/201731641. arXiv:1712.04505 [astro-ph.GA]

[CR31] Bugiani, L., Belli, S., Park, M., et al.: Active galactic nucleus feedback in quiescent galaxies at cosmic noon traced by ionized gas emission. Astrophys. J. **981**(1), 25 (2025). 10.3847/1538-4357/adaeaf. arXiv:2406.08547 [astro-ph.GA]

[CR32] Butler, K.M., van der Werf P.P., Topkaras, T., et al.: Molecular outflows in unobscured QSO hosts driven by star formation. Astrophys. J. **944**(2), 134 (2023). 10.3847/1538-4357/acad03. arXiv:2212.09675 [astro-ph.GA]

[CR33] Cano-Díaz, M., Maiolino, R., Marconi, A., et al.: Observational evidence of quasar feedback quenching star formation at high redshift. Astron. Astrophys. **537**, L8 (2012). 10.1051/0004-6361/201118358. arXiv:1112.3071

[CR34] Cappellari, M.: Full spectrum fitting with photometry in PPXF: stellar population versus dynamical masses, non-parametric star formation history and metallicity for 3200 LEGA-C galaxies at redshift . Mon. Not. R. Astron. Soc. **526**, 3273–3300 (2023). 10.1093/mnras/stad2597. arXiv:2208.14974

[CR35] Carnall, A.C., McLure, R.J., Dunlop, J.S., et al.: The VANDELS survey: the star-formation histories of massive quiescent galaxies at . Mon. Not. R. Astron. Soc. **490**(1), 417–439 (2019). 10.1093/mnras/stz2544. arXiv:1903.11082 [astro-ph.GA]

[CR36] Carnall, A.C., McLure, R.J., Dunlop, J.S., et al.: A massive quiescent galaxy at redshift 4.658. Nature **619**(7971), 716–719 (2023). 10.1038/s41586-023-06158-6. arXiv:2301.11413 [astro-ph.GA] PMC10371866 37216978

[CR37] Carnall, A.C., Cullen, F., McLure, R.J., et al.: The JWST EXCELS survey: too much, too young, too fast? Ultra-massive quiescent galaxies at . Mon. Not. R. Astron. Soc. **534**(1), 325–348 (2024). 10.1093/mnras/stae2092. arXiv:2405.02242 [astro-ph.GA]

[CR38] Carniani, S., Marconi, A., Maiolino, R., et al.: Fast outflows and star formation quenching in quasar host galaxies. Astron. Astrophys. **591**, A28 (2016). 10.1051/0004-6361/201528037. arXiv:1604.04290 [astro-ph.GA]

[CR39] Carniani, S., Marconi, A., Maiolino, R., et al.: AGN feedback on molecular gas reservoirs in quasars at . Astron. Astrophys. **605**, A105 (2017). 10.1051/0004-6361/201730672. arXiv:1706.08987

[CR40] Carniani, S., Venturi, G., Parlanti, E., et al.: JADES: the incidence rate and properties of galactic outflows in low-mass galaxies across . Astron. Astrophys. **685**, A99 (2024). 10.1051/0004-6361/202347230. arXiv:2306.11801 [astro-ph.GA]

[CR41] Catinella, B., Saintonge, A., Janowiecki, S., et al.: xGASS: total cold gas scaling relations and molecular-to-atomic gas ratios of galaxies in the local Universe. Mon. Not. R. Astron. Soc. **476**(1), 875–895 (2018). 10.1093/mnras/sty089. arXiv:1802.02373 [astro-ph.GA]

[CR42] Chandro-Gómez, Á., Lagos, C.D.P., Power, C., et al.: Unveiling the population of massive quenched galaxies at in the COLIBRE simulations – II. The role of AGN feedback and environment on their emergence. arXiv e-prints (2026). 10.48550/arXiv.2605.31052. arXiv:2605.31052 [astro-ph.GA]

[CR43] Chartas, G., Davidson, E., Brusa, M., et al.: Linking the small-scale relativistic winds and the large-scale molecular outflows in the lensed quasar HS 0810 + 2554. Mon. Not. R. Astron. Soc. **496**(1), 598–611 (2020). 10.1093/mnras/staa1534. arXiv:2005.13967 [astro-ph.GA]

[CR44] Chen, Y.M., Tremonti, C.A., Heckman, T.M., et al.: Absorption-line probes of the prevalence and properties of outflows in present-day star-forming galaxies. Astron. J. **140**, 445–461 (2010). 10.1088/0004-6256/140/2/445. arXiv:1003.5425

[CR45] Chen, Y.M., Shi, Y., Wild, V., et al.: Post-starburst galaxies in SDSS-IV MaNGA. Mon. Not. R. Astron. Soc. **489**(4), 5709–5722 (2019). 10.1093/mnras/stz2494. arXiv:1909.01658 [astro-ph.GA]

[CR46] Chen, Z., Fielding, D.B., Bryan, G.L.: The anatomy of a turbulent radiative mixing layer: insights from an analytic model with turbulent conduction and viscosity. Astrophys. J. **950**(2), 91 (2023). 10.3847/1538-4357/acc73f. arXiv:2211.01395 [astro-ph.GA]

[CR47] Chisholm, J., Tremonti, C.A., Leitherer, C., et al.: Shining a light on galactic outflows: photoionized outflows. Mon. Not. R. Astron. Soc. **457**, 3133–3161 (2016). 10.1093/mnras/stw178. arXiv:1601.05090

[CR48] Cicone, C., Maiolino, R., Sturm, E., et al.: Massive molecular outflows and evidence for AGN feedback from CO observations. Astron. Astrophys. **562**, A21 (2014). 10.1051/0004-6361/201322464. arXiv:1311.2595

[CR49] Concas, A., Popesso, P., Brusa, M., et al.: Two-face(s): ionized and neutral gas winds in the local Universe. Astron. Astrophys. **622**, A188 (2019). 10.1051/0004-6361/201732152

[CR50] Concas, A., Maiolino, R., Curti, M., et al.: Being KLEVER at cosmic noon: ionized gas outflows are inconspicuous in low-mass star-forming galaxies but prominent in massive AGN hosts. Mon. Not. R. Astron. Soc. **513**(2), 2535–2562 (2022). 10.1093/mnras/stac1026. arXiv:2203.11958 [astro-ph.GA]

[CR51] Couto, G.S., Storchi-Bergmann, T.: The interplay between radio AGN activity and their host galaxies. Galaxies **11**(2), 47. (2023). 10.3390/galaxies11020047 arXiv:2303.12033 [astro-ph.GA]

[CR52] Cox, D.P.: The three-phase interstellar medium revisited. Annu. Rev. Astron. Astrophys. **43**(1), 337–385 (2005). 10.1146/annurev.astro.43.072103.150615

[CR53] Crain, R.A., van de Voort, F.: Hydrodynamical simulations of the galaxy population: enduring successes and outstanding challenges. Annu. Rev. Astron. Astrophys. **61**, 473–515 (2023). 10.1146/annurev-astro-041923-043618. arXiv:2309.17075 [astro-ph.GA]

[CR54] Crain, R.A., Schaye, J., Bower, R.G., et al.: The EAGLE simulations of galaxy formation: calibration of subgrid physics and model variations. Mon. Not. R. Astron. Soc. **450**(2), 1937–1961 (2015). 10.1093/mnras/stv725. arXiv:1501.01311 [astro-ph.GA]

[CR55] Cresci, G., Mainieri, V., Brusa, M., et al.: Blowin’ in the wind: both “negative” and “positive” feedback in an obscured high-z quasar. Astrophys. J. **799**, 82 (2015). 10.1088/0004-637X/799/1/82. arXiv:1411.4208

[CR56] Croton, D.J., Springel, V., White, S.D.M., et al.: The many lives of active galactic nuclei: cooling flows, black holes and the luminosities and colours of galaxies. Mon. Not. R. Astron. Soc. **365**, 11–28 (2006). 10.1111/j.1365-2966.2005.09675.x. arXiv:astro-ph/0508046

[CR57] Davé, R., Anglés-Alcázar, D., Narayanan, D., et al.: SIMBA: cosmological simulations with black hole growth and feedback. Mon. Not. R. Astron. Soc. **486**(2), 2827–2849 (2019). 10.1093/mnras/stz937. arXiv:1901.10203 [astro-ph.GA]

[CR58] Davies, R.L., Förster Schreiber, N.M. Übler, H., et al.: Kiloparsec scale properties of star formation driven outflows at in the SINS/zC-SINF AO survey. Astrophys. J. **873**(2), 122 (2019). 10.3847/1538-4357/ab06f1. arXiv:1808.10700 [astro-ph.GA]

[CR59] Davies, R.L., Förster Schreiber, N.M., Lutz, D., et al.: From nuclear to circumgalactic: zooming in on AGN-driven outflows at with SINFONI. Astrophys. J. **894**(1), 28 (2020). 10.3847/1538-4357/ab86ad

[CR60] Davies, R., Shimizu, T., Pereira-Santaella, M., et al.: GATOS: missing molecular gas in the outflow of NGC 5728 revealed by JWST. Astron. Astrophys. **689**, A263 (2024a). 10.1051/0004-6361/202449875. arXiv:2406.17072 [astro-ph.GA]

[CR61] Davies, R.L., Belli, S., Park, M., et al.: JWST reveals widespread AGN-driven neutral gas outflows in massive galaxies. Mon. Not. R. Astron. Soc. **528**(3), 4976–4992 (2024b). 10.1093/mnras/stae327. arXiv:2310.17939 [astro-ph.GA]

[CR62] Davies, R.L., Fisher, D.B., Herrera-Camus, R., et al.: Multiphase images of a powerful supernova-driven wind in the early Universe. Mon. Not. R. Astron. Soc. **549**(3), stag874 (2026). 10.1093/mnras/stag874

[CR63] Davis, T.A., Krajnović, D., McDermid, R.M., et al.: Gemini GMOS and WHT SAURON integral-field spectrograph observations of the AGN-driven outflow in NGC 1266. Mon. Not. R. Astron. Soc. **426**(2), 1574–1590 (2012). 10.1111/j.1365-2966.2012.21770.x. arXiv:1207.5799 [astro-ph.CO]

[CR64] de Graaff, A., Setton, D.J., Brammer, G., et al.: Efficient formation of a massive quiescent galaxy at redshift 4.9. Nat. Astron. **9**, 280–292 (2025). 10.1038/s41550-024-02424-3. arXiv:2404.05683 [astro-ph.GA] PMC11842275 39990236

[CR65] Dekel, A., Burkert, A.: Wet disc contraction to galactic blue nuggets and quenching to red nuggets. Mon. Not. R. Astron. Soc. **438**(2), 1870–1879 (2014). 10.1093/mnras/stt2331. arXiv:1310.1074 [astro-ph.CO]

[CR66] D’Eugenio, C., Daddi, E., Gobat, R., et al.: The typical massive quiescent galaxy at is a post-starburst. Astrophys. J. Lett. **892**(1), L2 (2020). 10.3847/2041-8213/ab7a96. arXiv:2003.04342 [astro-ph.GA]

[CR67] D’Eugenio, F., Pérez-González, P.G., Maiolino, R., et al.: A fast-rotator post-starburst galaxy quenched by supermassive black-hole feedback at . Nat. Astron. **8**, 1443–1456 (2024). 10.1038/s41550-024-02345-1. arXiv:2308.06317 [astro-ph.GA]

[CR68] Di Matteo, T., Springel, V., Hernquist, L.: Energy input from quasars regulates the growth and activity of black holes and their host galaxies. Nature **433**, 604–607 (2005). 10.1038/nature03335. arXiv:astro-ph/0502199 15703739

[CR69] Dolag, K., Remus, R.S., Valenzuela, L.M., et al.: Encyclopedia magneticum: scaling relations from cosmic dawn to present day. arXiv e-prints (2025). 10.48550/arXiv.2504.01061. arXiv:2504.01061 [astro-ph.CO]

[CR70] Draine, B.T.: Physics of the Interstellar and Intergalactic Medium (2011)

[CR71] Dressler, A., Gunn, J.E.: Spectroscopy of galaxies in distant clusters. II. The population of the 3C 295 cluster. Astrophys. J. **270**, 7–19 (1983). 10.1086/161093

[CR72] Edward, A.H., Antwi-Danso, J., Muzzin, A., et al.: MAGAZ3NE: spatially resolved ages and chemical abundances of ultra-massive quiescent galaxies at using JWST/NIRSpec IFU. arXiv e-prints (2026). arXiv:2605.27555 [astro-ph.GA]

[CR73] Erb, D.K.: Feedback in low-mass galaxies in the early Universe. Nature **523**, 169–176 (2015) 10.1038/nature14454. arXiv:1507.02374 26156371

[CR74] Farber, R.J., Gronke, M.: The survival of multiphase dusty clouds in hot winds. Mon. Not. R. Astron. Soc. **510**(1), 551–567 (2022). 10.1093/mnras/stab3412. arXiv:2107.07991 [astro-ph.GA]

[CR75] Fernández-Ontiveros, J.A., Spinoglio, L., Pereira-Santaella, M., et al.: Wild is the wind from low-luminosity AGN: a jet-driven gas bubble blowing out a massive CO-dark outflow in ESO 420-G13. arXiv e-prints (2026). 10.48550/arXiv.2604.06305. arXiv:2604.06305 [astro-ph.GA]

[CR76] Ferrière, K.M.: The interstellar environment of our galaxy. Rev. Mod. Phys. **73**(4), 1031–1066 (2001). 10.1103/RevModPhys.73.1031. arXiv:astro-ph/0106359 [astro-ph]

[CR77] Feruglio, C., Fiore, F., Carniani, S., et al.: The multi-phase winds of Markarian 231: from the hot, nuclear, ultra-fast wind to the galaxy-scale, molecular outflow. Astron. Astrophys. **583**, A99 (2015). 10.1051/0004-6361/201526020. arXiv:1503.01481 [astro-ph.GA]

[CR78] Field, G.B., Goldsmith, D.W., Habing, H.J.: Cosmic-ray heating of the interstellar gas. Astrophys. J. Lett. **155**, L149 (1969). 10.1086/180324

[CR79] Fielding, D.B., Bryan, G.L.: The structure of multiphase galactic winds. Astrophys. J. **924**(2), 82 (2022). 10.3847/1538-4357/ac2f41. arXiv:2108.05355 [astro-ph.GA]

[CR80] Fielding, D.B., Ostriker, E.C., Bryan, G.L., et al.: Multiphase gas and the fractal nature of radiative turbulent mixing layers. Astrophys. J. Lett. **894**(2), L24 (2020). 10.3847/2041-8213/ab8d2c. arXiv:2003.08390 [astro-ph.GA]

[CR81] Fiore, F., Feruglio, C., Shankar, F., et al.: AGN wind scaling relations and the co-evolution of black holes and galaxies. Astron. Astrophys. **601**, A143 (2017). 10.1051/0004-6361/201629478. arXiv:1702.04507 [astro-ph.GA]

[CR82] Fluetsch, A., Maiolino, R., Carniani, S., et al.: Cold molecular outflows in the local Universe and their feedback effect on galaxies. Mon. Not. R. Astron. Soc. **483**(4), 4586–4614 (2019). 10.1093/mnras/sty3449. arXiv:1805.05352 [astro-ph.GA]

[CR83] Forrest, B., Tran, K.V.H., Broussard, A., et al.: ZFOURGE: using composite spectral energy distributions to characterize galaxy populations at . Astrophys. J. **863**(2), 131 (2018). 10.3847/1538-4357/aad232. arXiv:1807.03785 [astro-ph.GA]

[CR84] Forrest, B., Muzzin, A., Marchesini, D., et al.: A massive and evolved slow-rotating galaxy in the early universe. Nat. Astron. **10**, 1232–1240 (2026). 10.1038/s41550-026-02855-0. https://www.nature.com/articles/s41550-026-02855-0

[CR85] Förster Schreiber, N.M., Wuyts, S.: Star-forming galaxies at cosmic noon. Annu. Rev. Astron. Astrophys. **58**, 661–725 (2020). 10.1146/annurev-astro-032620-021910. arXiv:2010.10171 [astro-ph.GA]

[CR86] Förster Schreiber, N.M., Genzel, R., Newman, S.F., et al.: The Sins/zC-Sinf Survey of galaxy kinematics: evidence for powerful active galactic nucleus-driven nuclear outflows in massive star-forming galaxies. Astrophys. J. **787**, 38 (2014). 10.1088/0004-637X/787/1/38. arXiv:1311.2596

[CR87] Förster Schreiber, N.M., Übler, H., Davies, R.L., et al.: The KMOS^3D^ survey: demographics and properties of galactic outflows at . Astrophys. J. **875**(1), 21 (2019). 10.3847/1538-4357/ab0ca2. arXiv:1807.04738 [astro-ph.GA]

[CR88] Franx, M., van Dokkum, P.G., Förster Schreiber, N.M., et al.: Structure and star formation in galaxies out to : evidence for surface density dependent evolution and upsizing. Astrophys. J. **688**(2), 770–788 (2008). 10.1086/592431. arXiv:0808.2642 [astro-ph]

[CR89] Freeman, W.R., Siana, B., Kriek, M., et al.: The MOSDEF Survey: broad emission lines at . Astrophys. J. **873**(2), 102 (2019). 10.3847/1538-4357/ab0655

[CR90] French, K.D.: Evolution through the post-starburst phase: using post-starburst galaxies as laboratories for understanding the processes that drive galaxy evolution. Publ. Astron. Soc. Pac. **133**(1025), 072001 (2021). 10.1088/1538-3873/ac0a59. arXiv:2106.05982 [astro-ph.GA]

[CR91] French, K.D., Yang, Y., Zabludoff, A.I., et al.: Clocking the evolution of post-starburst galaxies: methods and first results. Astrophys. J. **862**(1), 2 (2018). 10.3847/1538-4357/aacb2d10.48550/arXiv.1806.03301. arXiv:1806.03301 [astro-ph.GA]

[CR92] French, K.D., Nyland, K., Patil, P., et al.: Radio variability in recently quenched galaxies: the impact of tidal disruption event or active galactic nucleus-driven outflows. Astrophys. J. **992**(1), 123 (2025). 10.3847/1538-4357/adff7a. arXiv:2507.21838 [astro-ph.GA]

[CR93] Genzel, R., Newman, S., Jones, T., et al.: The Sins survey of galaxy kinematics: properties of the giant star-forming clumps. Astrophys. J. **733**, 101 (2011). 10.1088/0004-637X/733/2/101. arXiv:1011.5360

[CR94] Genzel, R., Förster Schreiber, N.M., Rosario, D., et al.: Evidence for wide-spread active galactic nucleus-driven outflows in the most massive star-forming galaxies. Astrophys. J. **796**, 7 (2014). 10.1088/0004-637X/796/1/7. arXiv:1406.0183

[CR95] Girichidis, P., Offner, S.S.R., Kritsuk, A.G., et al.: Physical processes in star formation. Space Sci. Rev. **216**(4), 68 (2020). 10.1007/s11214-020-00693-8. arXiv:2005.06472 [astro-ph.GA]

[CR96] Glazebrook, K., Nanayakkara, T., Schreiber, C., et al.: A massive galaxy that formed its stars at . Nature **628**(8007), 277–281 (2024). 10.1038/s41586-024-07191-9. arXiv:2308.05606 [astro-ph.GA] 38354832

[CR97] Goto, T.: A catalogue of local E + A (post-starburst) galaxies selected from the Sloan Digital Sky Survey Data Release 5. Mon. Not. R. Astron. Soc. **381**(1), 187–193 (2007). 10.1111/j.1365-2966.2007.12227.x. arXiv:0801.1106 [astro-ph]

[CR98] Gronke, M., Oh, S.P.: The growth and entrainment of cold gas in a hot wind. Mon. Not. R. Astron. Soc. **480**(1), L111–L115 (2018). 10.1093/mnrasl/sly131. arXiv:1806.02728 [astro-ph.GA]

[CR99] Harrison, C.M., Ramos Almeida, C.: Observational tests of active galactic nuclei feedback: an overview of approaches and interpretation. Galaxies **12**(2), 17 (2024). 10.3390/galaxies12020017. arXiv:2404.08050 [astro-ph.GA]

[CR100] Harrison, C.M., Alexander, D.M., Swinbank, A.M., et al.: Energetic galaxy-wide outflows in high-redshift ultraluminous infrared galaxies hosting AGN activity. Mon. Not. R. Astron. Soc. **426**, 1073–1096 (2012). 10.1111/j.1365-2966.2012.21723.x. arXiv:1205.1801

[CR101] Harrison, C.M., Alexander, D.M., Mullaney, J.R., et al.: The KMOS AGN Survey at High Redshift (KASHz): the prevalence and drivers of ionized outflows in the host galaxies of X-ray AGN. Mon. Not. R. Astron. Soc. **456**(2), 1195–1220 (2016). 10.1093/mnras/stv2727. arXiv:1511.00008 [astro-ph.GA]

[CR102] Heckman, T.M., Lehnert, M.D., Strickland, D.K., et al.: Absorption-line probes of gas and dust in galactic superwinds. Astrophys. J. Suppl. Ser. **129**, 493–516 (2000). 10.1086/313421. arXiv:astro-ph/0002526

[CR103] Hernández-Yévenes, J., Fisher, D.B., Mazzilli Ciraulo, B., et al.: The GECKOS survey: resolving the molecular and ionised gas in the galactic outflow of ESO∼484-036. arXiv e-prints (2026). 10.48550/arXiv.2604.14546. arXiv:2604.14546 [astro-ph.GA]

[CR104] Herrera-Camus, R., Tacconi, L., Genzel, R., et al.: Molecular and ionized gas phases of an AGN-driven outflow in a typical massive galaxy at . Astrophys. J. **871**, 37 (2019). 10.3847/1538-4357/aaf6a7. arXiv:1807.07074

[CR105] Hewitt, G., Almaini, O., Maltby, D., et al.: No hidden monsters: probing recently-quenched galaxies for obscured AGN with JWST-PRIMER MIRI and NIRCam. arXiv e-prints (2026). 10.48550/arXiv.2606.20800. arXiv:2606.20800 [astro-ph.GA]

[CR106] Hopkins, P.F., Hernquist, L., Cox, T.J., et al.: A unified, merger-driven model of the origin of starbursts, quasars, the cosmic X-ray background, supermassive black holes, and galaxy spheroids. Astrophys. J. Suppl. Ser. **163**, 1–49 (2006). 10.1086/499298. arXiv:astro-ph/0506398

[CR107] Ilbert, O., McCracken, H.J., Le Fèvre, O., et al.: Mass assembly in quiescent and star-forming galaxies since from UltraVISTA. Astron. Astrophys. **556**, A55 (2013). 10.1051/0004-6361/201321100. arXiv:1301.3157 [astro-ph.CO]

[CR108] Ilha, G.S., Riffel, R.A., Ricci, T.V., et al.: Active galactic nuclei signatures in Red Geyser galaxies from Gemini GMOS-IFU observations. Mon. Not. R. Astron. Soc. **516**(1), 1442–1461 (2022). 10.1093/mnras/stac2233. arXiv:2208.03383 [astro-ph.GA]

[CR109] Ji, S., Oh, S.P., Masterson, P.: Simulations of radiative turbulent mixing layers. Mon. Not. R. Astron. Soc. **487**(1), 737–754 (2019). 10.1093/mnras/stz1248. arXiv:1809.09101 [astro-ph.GA]

[CR110] Johnson, B.D., Leja, J., Conroy, C., et al.: Stellar population inference with prospector. Astrophys. J. Suppl. Ser. **254**(2), 22 (2021). 10.3847/1538-4365/abef67. arXiv:2012.01426 [astro-ph.GA]

[CR111] Kakkad, D., Mainieri, V., Vietri, G., et al.: SUPER VII. Morphology and kinematics of H emission in AGN host galaxies at cosmic noon using SINFONI. Mon. Not. R. Astron. Soc. **520**(4), 5783–5802 (2023). 10.1093/mnras/stad439. arXiv:2302.03039 [astro-ph.GA]

[CR112] Kehoe, E., Shapley, A.E., Schreiber, N.M.F., et al.: The first combined H and rest-UV spectroscopic probe of galactic outflows at High Redshift. Astrophys. J. **976**(1), 28 (2024). 10.3847/1538-4357/ad7ebb. arXiv:2406.07621 [astro-ph.GA]

[CR113] Kehoe, E., Shapley, A.E., Carnall, A.C., et al.: Unprecedented constraints on gas flows at high redshift using deep JWST/NIRSpec observations from the LyC22, EXCELS, and AURORA surveys. arXiv e-prints (2026). 10.48550/arXiv.2606.12686. arXiv:2606.12686 [astro-ph.GA]

[CR114] Kim, C.G., Ostriker, E.C., Somerville, R.S., et al.: First results from SMAUG: characterization of multiphase galactic outflows from a suite of local star-forming galactic disk simulations. Astrophys. J. **900**(1), 61 (2020). 10.3847/1538-4357/aba962. arXiv:2006.16315 [astro-ph.GA]

[CR115] Kimmig, L.C., Remus, R.S., Seidel, B., et al.: Blowing out the candle: how to quench galaxies at High Redshift—an ensemble of rapid starbursts, AGN feedback, and environment. Astrophys. J. **979**(1), 15 (2025). 10.3847/1538-4357/ad9472. arXiv:2310.16085 [astro-ph.GA]

[CR116] King, A., Pounds, K.: Powerful outflows and feedback from active galactic nuclei. Annu. Rev. Astron. Astrophys. **53**, 115–154 (2015). 10.1146/annurev-astro-082214-122316. arXiv:1503.05206

[CR117] Kornei, K.A., Shapley, A.E., Martin, C.L., et al.: The properties and prevalence of galactic outflows at in the extended groth strip. Astrophys. J. **758**, 135 (2012). 10.1088/0004-637X/758/2/135. arXiv:1205.0812

[CR118] Lagos, C.D.P., Valentino, F., Wright, R.J., et al.: The diverse star formation histories of early massive, quenched galaxies in modern galaxy formation simulations. Mon. Not. R. Astron. Soc. **536**(3), 2324–2354 (2025). 10.1093/mnras/stae2626. arXiv:2409.16916 [astro-ph.GA]

[CR119] Lang, P., Wuyts, S., Somerville, R.S., et al.: Bulge growth and quenching since in CANDELS/3D-HST. Astrophys. J. **788**, 11 (2014). 10.1088/0004-637X/788/1/11. arXiv:1402.0866

[CR120] Langan, I., Popping, G., Ginolfi, M., et al.: A search for cold molecular outflows in cosmic-noon galaxies. Astron. Astrophys. **705**, A209 (2026). 10.1051/0004-6361/202554297. arXiv:2511.16733 [astro-ph.GA]

[CR121] Larson, R.B., Tinsley, B.M., Caldwell, C.N.: The evolution of disk galaxies and the origin of S0 galaxies. Astrophys. J. **237**, 692–707 (1980). 10.1086/157917

[CR122] Leja, J., Carnall, A.C., Johnson, B.D., et al.: How to measure galaxy star formation histories. II. Nonparametric models. Astrophys. J. **876**(1), 3 (2019). 10.3847/1538-4357/ab133c. arXiv:1811.03637 [astro-ph.GA]

[CR123] Leroy, A.K., Bolatto, A.D., Ostriker, E.C., et al.: ALMA reveals the molecular medium fueling the nearest nuclear starburst. Astrophys. J. **801**, 25 (2015). 10.1088/0004-637X/801/1/25. arXiv:1411.2836

[CR124] Leung, G.C.K., Coil, A.L., Aird, J., et al.: The MOSDEF Survey: a census of AGN-driven ionized outflows at . Astrophys. J. **886**(1), 11 (2019). 10.3847/1538-4357/ab4a7c

[CR125] Liboni, C., Belli, S., Bugiani, L., et al.: Probing neutral outflows in galaxies using JWST observations of Ca II H and K absorption lines. Astron. Astrophys. **705**, A125 (2026). 10.1051/0004-6361/202555845. arXiv:2506.05470 [astro-ph.GA]

[CR126] Llerena, M., Amorín, R., Pentericci, L., et al.: Ionized gas kinematics and chemical abundances of low-mass star-forming galaxies at . Astron. Astrophys. **676**, A53 (2023). 10.1051/0004-6361/202346232

[CR127] Maltby, D.T., Almaini, O., McLure, R.J., et al.: High-velocity outflows in massive post-starburst galaxies at . Mon. Not. R. Astron. Soc. **489**(1), 1139–1151 (2019). 10.1093/mnras/stz2211. arXiv:1908.02766 [astro-ph.GA]

[CR128] Man, A.W.S., Zabl, J., Brammer, G.B., et al.: An exquisitely deep view of quenching galaxies through the gravitational lens: stellar population, morphology, and ionized gas. Astrophys. J. **919**(1), 20 (2021). 10.3847/1538-4357/ac0ae3. arXiv:2106.08338 [astro-ph.GA]

[CR129] Marasco, A., Belfiore, F., Cresci, G., et al.: Shaken, but not expelled: gentle baryonic feedback from nearby starburst dwarf galaxies. Astron. Astrophys. **670**, A92 (2023). 10.1051/0004-6361/202244895. arXiv:2209.02726 [astro-ph.GA]

[CR130] Martin, C.L.: Mapping large-scale gaseous outflows in ultraluminous galaxies with Keck II ESI spectra: variations in outflow velocity with galactic mass. Astrophys. J. **621**, 227–245 (2005). 10.1086/427277. arXiv:astro-ph/0410247

[CR131] Martin, C.L.: Mapping large-scale gaseous outflows in ultraluminous infrared galaxies with Keck II ESI spectra: spatial extent of the outflow. Astrophys. J. **647**(1), 222–243 (2006). 10.1086/504886. arXiv:astro-ph/0604173 [astro-ph]

[CR132] Mazzilli Ciraulo, B., Fisher, D.B., Elliott, R., et al.: The GECKOS Survey: resolved, multiphase observations of mass loading and gas density in the galactic wind of NGC 4666. Mon. Not. R. Astron. Soc. **544**(4), 3290–3311 (2025). 10.1093/mnras/staf1875. arXiv:2509.17560 [astro-ph.GA]

[CR133] McKee, C.F., Ostriker, J.P.: A theory of the interstellar medium - three components regulated by supernova explosions in an inhomogeneous substrate. Astrophys. J. **218**, 148–169 (1977). 10.1086/155667

[CR134] McQuinn, K.B.W., Skillman, E.D., Heilman, T.N., et al.: Galactic outflows, star formation histories, and time-scales in starburst dwarf galaxies from STARBIRDS. Mon. Not. R. Astron. Soc. **477**(3), 3164–3177 (2018). 10.1093/mnras/sty839. arXiv:1705.04692 [astro-ph.GA]

[CR135] Mitchell, P.D., Schaye, J., Bower, R.G., et al.: Galactic outflow rates in the EAGLE simulations. Mon. Not. R. Astron. Soc. **494**(3), 3971–3997 (2020). 10.1093/mnras/staa938. arXiv:1910.09566 [astro-ph.GA]

[CR136] Moghni, A., Roy, N., Heckman, T.M., et al.: Galactic rain: cool gas inflows in Red Geyser galaxies and their connection to active galactic nuclei activity and interactions. Astrophys. J. **1003**(1), 31 (2026). 10.3847/1538-4357/ae5f90. arXiv:2604.13195 [astro-ph.GA]

[CR137] Moretti, L., Belli, S., Rudie, G.C., et al.: Empirical calibration of Na I D and other absorption lines as tracers of high-redshift neutral outflows. Astron. Astrophys. **707**, A146 (2026). 10.1051/0004-6361/202556336. arXiv:2507.07160 [astro-ph.GA]

[CR138] Murray, N., Martin, C.L., Quataert, E., et al.: The ionization state of sodium in galactic winds. Astrophys. J. **660**(1), 211–220 (2007). 10.1086/512660. arXiv:astro-ph/0609213 [astro-ph]

[CR139] Naab, T., Ostriker, J.P.: Theoretical challenges in galaxy formation. Annu. Rev. Astron. Astrophys. **55**(1), 59–109 (2017). 10.1146/annurev-astro-081913-040019. arXiv:1612.06891 [astro-ph.GA]

[CR140] Nanayakkara, T., Glazebrook, K., Schreiber, C., et al.: The formation histories of massive and quiescent galaxies in the Universe. Astrophys. J. **981**(1), 78 (2025). 10.3847/1538-4357/ada6ac. arXiv:2410.02076 [astro-ph.GA]

[CR141] Nelson, D., Pillepich, A., Springel, V., et al.: First results from the TNG50 simulation: galactic outflows driven by supernovae and black hole feedback. Mon. Not. R. Astron. Soc. **490**(3), 3234–3261 (2019). 10.1093/mnras/stz2306. arXiv:1902.05554 [astro-ph.GA]

[CR142] Nesvadba, N.P.H., Lehnert, M.D., De Breuck, C., et al.: Evidence for powerful AGN winds at high redshift: dynamics of galactic outflows in radio galaxies during the “Quasar Era”. Astron. Astrophys. **491**(2), 407–424 (2008). 10.1051/0004-6361:200810346. arXiv:0809.5171 [astro-ph]

[CR143] Newman, S.F., Genzel, R., Förster-Schreiber, N.M., et al.: The SINS/zC-SINF Survey of galaxy kinematics: outflow properties. Astrophys. J. **761**, 43 (2012a). 10.1088/0004-637X/761/1/43. arXiv:1207.5897

[CR144] Newman, S.F., Shapiro Griffin, K., Genzel, R., et al.: Shocked superwinds from the clumpy star-forming galaxy, ZC406690. Astrophys. J. **752**, 111 (2012b). 10.1088/0004-637X/752/2/111. arXiv:1204.4727 [astro-ph.CO]

[CR145] Oosterloo, T., Raymond Oonk, J.B., Morganti, R., et al.: Properties of the molecular gas in the fast outflow in the Seyfert galaxy IC 5063. Astron. Astrophys. **608**, A38 (2017). 10.1051/0004-6361/201731781. arXiv:1710.01570 [astro-ph.GA]

[CR146] Park, M., Belli, S., Conroy, C., et al.: Rapid quenching of galaxies at cosmic noon. Astrophys. J. **953**(1), 119 (2023). 10.3847/1538-4357/acd54a

[CR147] Park, M., Belli, S., Conroy, C., et al.: Widespread rapid quenching at cosmic noon revealed by JWST deep spectroscopy. Astrophys. J. **976**(1), 72 (2024). 10.3847/1538-4357/ad7e15. arXiv:2404.17945 [astro-ph.GA]

[CR148] Patil, P., Rowlands, K., Alatalo, K., et al.: Tracing radio AGN-driven quenching in post-starburst galaxies at cosmic noon. arXiv e-prints (2026). 10.48550/arXiv.2605.11101. arXiv:2605.11101 [astro-ph.GA]

[CR149] Peng, Y., Lilly, S.J., Kovač, K., et al.: Mass and environment as drivers of galaxy evolution in SDSS and zCOSMOS and the origin of the Schechter function. Astrophys. J. **721**, 193–221 (2010). 10.1088/0004-637X/721/1/193. arXiv:1003.4747 [astro-ph.CO]

[CR150] Peng, Y., Maiolino, R., Cochrane, R.: Strangulation as the primary mechanism for shutting down star formation in galaxies. Nature **521**(7551), 192–195 (2015). 10.1038/nature14439. arXiv:1505.03143 [astro-ph.GA] 25971510

[CR151] Peroux, C., Merloni, A., Liske, J., et al.: Transform our understanding of the baryon cycle with high-resolution quasar spectroscopy (ByCycle). Messenger **190**, 42–45 (2023). 10.18727/0722-6691/5311

[CR152] Pessa, I., Wisotzki, L., Urrutia, T., et al.: First statistical constraints on galactic-scale outflow properties traced by their extended Mg II emission with MUSE. Astron. Astrophys. **708**, A214 (2026). 10.1051/0004-6361/202557875. arXiv:2602.11280 [astro-ph.GA]

[CR153] Pettini, M., Shapley, A.E., Steidel, C.C., et al.: The rest-frame optical spectra of Lyman break galaxies: star formation, extinction, abundances, and kinematics. Astrophys. J. **554**(2), 981–1000 (2001). 10.1086/321403. arXiv:astro-ph/0102456 [astro-ph]

[CR154] Pillepich, A., Springel, V., Nelson, D., et al.: Simulating galaxy formation with the IllustrisTNG model. Mon. Not. R. Astron. Soc. **473**(3), 4077–4106 (2018). 10.1093/mnras/stx2656. arXiv:1703.02970 [astro-ph.GA]

[CR155] Quintero, A.D., Hogg, D.W., Blanton, M.R., et al.: Selection and photometric properties of K + A galaxies. Astrophys. J. **602**(1), 190–199 (2004). 10.1086/380601. arXiv:astro-ph/0307074 [astro-ph]

[CR156] Rathjen, T.E., Naab, T., Walch, S., et al.: SILCC - VII. Gas kinematics and multiphase outflows of the simulated ISM at high gas surface densities. Mon. Not. R. Astron. Soc. **522**(2), 1843–1862 (2023). 10.1093/mnras/stad1104. arXiv:2211.15419 [astro-ph.GA]

[CR157] Revalski, M., Meena, B., Martinez, F., et al.: Quantifying feedback from narrow line region outflows in nearby active galaxies. III. Results for the Seyfert 2 galaxies Markarian 3, Markarian 78, and NGC 1068. Astrophys. J. **910**(2), 139 (2021). 10.3847/1538-4357/abdcad. arXiv:2101.06270 [astro-ph.GA]

[CR158] Richie, H.M., Schneider, E.E.: Dust evolution in simulated multiphase galactic outflows. Astrophys. J. **996**(1), 17 (2026). 10.3847/1538-4357/ae12e8. arXiv:2505.11734 [astro-ph.GA]

[CR159] Richings, A.J., Faucher-Giguère, C.A.: Radiative cooling of swept-up gas in AGN-driven galactic winds and its implications for molecular outflows. Mon. Not. R. Astron. Soc. **478**(3), 3100–3119 (2018). 10.1093/mnras/sty1285. arXiv:1710.09433 [astro-ph.GA]

[CR160] Roberts-Borsani, G.W.: Observational constraints on the multiphase nature of outflows using large spectroscopic surveys at . Mon. Not. R. Astron. Soc. **494**(3), 4266–4278 (2020). 10.1093/mnras/staa1006. arXiv:2004.06116 [astro-ph.GA]

[CR161] Roberts-Borsani, G.W., Saintonge, A.: The prevalence and properties of cold gas inflows and outflows around galaxies in the local Universe. Mon. Not. R. Astron. Soc. **482**(3), 4111–4145 (2019). 10.1093/mnras/sty2824

[CR162] Rowlands, K., Wild, V., Bourne, N., et al.: Galaxy and Mass Assembly (GAMA): the mechanisms for quiescent galaxy formation at . Mon. Not. R. Astron. Soc. **473**(1), 1168–1185 (2018). 10.1093/mnras/stx1903. arXiv:1707.07989 [astro-ph.GA]

[CR163] Roy, N., Bundy, K., Rubin, K.H.R., et al.: Signatures of inflowing gas in Red Geyser galaxies hosting radio active galactic nuclei. Astrophys. J. **919**(2), 145 (2021). 10.3847/1538-4357/ac0f74. arXiv:2106.14901 [astro-ph.GA]

[CR164] Rubin, K.H.R., Juarez, C., Cooksey, K.L., et al.: On the kinematics of cold, metal-enriched galactic fountain flows in nearby star-forming galaxies. Astrophys. J. **936**(2), 171 (2022). 10.3847/1538-4357/ac7b88

[CR165] Rupke, D.S.N., Veilleux, S.: Spatially extended Na I D resonant emission and absorption in the galactic wind of the nearby infrared-luminous quasar F05189-2524. Astrophys. J. **801**(2), 126 (2015). 10.1088/0004-637X/801/2/126. arXiv:1411.3744 [astro-ph.GA]

[CR166] Rupke, D.S., Veilleux, S., Sanders, D.B.: Outflows in infrared-luminous starbursts at . I. Sample, Na I D spectra, and profile fitting. Astrophys. J. Suppl. Ser. **160**(1), 87–114 (2005a). 10.1086/432886. arXiv:astro-ph/0506610 [astro-ph]

[CR167] Rupke, D.S., Veilleux, S., Sanders, D.B.: Outflows in infrared-luminous starbursts at . II. Analysis and discussion. Astrophys. J. Suppl. Ser. **160**, 115–148 (2005b). 10.1086/432889. arXiv:astro-ph/0506611

[CR168] Rupke, D.S.N., Gültekin, K., Veilleux, S.: Quasar-mode feedback in nearby type 1 quasars: ubiquitous kiloparsec-scale outflows and correlations with black hole properties. Astrophys. J. **850**(1), 40 (2017). 10.3847/1538-4357/aa94d1. arXiv:1708.05139 [astro-ph.GA]

[CR169] Saintonge, A., Catinella, B.: The cold interstellar medium of galaxies in the local Universe. Annu. Rev. Astron. Astrophys. **60**, 319–361 (2022). 10.1146/annurev-astro-021022-043545. arXiv:2202.00690 [astro-ph.GA]

[CR170] Savage, B.D., Sembach, K.R.: Interstellar abundances from absorption-line observations with the Hubble Space Telescope. Annu. Rev. Astron. Astrophys. **34**, 279–330 (1996). 10.1146/annurev.astro.34.1.279

[CR171] Schaye, J., Crain, R.A., Bower, R.G., et al.: The EAGLE project: simulating the evolution and assembly of galaxies and their environments. Mon. Not. R. Astron. Soc. **446**(1), 521–554 (2015). 10.1093/mnras/stu2058. arXiv:1407.7040 [astro-ph.GA]

[CR172] Schneider, E.E., Robertson, B.E., Thompson, T.A.: Production of cool gas in thermally driven outflows. Astrophys. J. **862**(1), 56 (2018). 10.3847/1538-4357/aacce1. arXiv:1803.01005 [astro-ph.GA]

[CR173] Scholtz, J., Harrison, C.M., Rosario, D.J., et al.: The impact of ionized outflows from quasars is not through instantaneous in situ quenching: the evidence from ALMA and VLT/SINFONI. Mon. Not. R. Astron. Soc. **505**(4), 5469–5487 (2021). 10.1093/mnras/stab1631. arXiv:2106.05277 [astro-ph.GA]

[CR174] Scholtz, J., D’Eugenio, F., Maiolino, R., et al.: Measurement of the gas consumption history of a massive quiescent galaxy. Nat. Astron. **10**, 431–439 (2026). 10.1038/s41550-025-02751-z

[CR175] Shapley, A.E., Steidel, C.C., Pettini, M., et al.: Rest-frame ultraviolet spectra of Lyman break galaxies. Astrophys. J. **588**, 65–89 (2003). 10.1086/373922. arXiv:astro-ph/0301230

[CR176] Shih, H.Y., Rupke, D.S.N.: The complex structure of the multi-phase galactic wind in a starburst merger. Astrophys. J. **724**(2), 1430–1440 (2010). 10.1088/0004-637X/724/2/1430. arXiv:1009.6020 [astro-ph.CO]

[CR177] Skarbinski, M., Rowlands, K., Alatalo, K., et al.: The JWST EXCELS survey: insights into the nature of quenching at cosmic noon. Astrophys. J. **1000**(2), 191 (2026). 10.3847/1538-4357/ae459d. arXiv:2509.18278 [astro-ph.GA]

[CR178] Slob, M., Kriek, M., Beverage, A.G., et al.: The JWST-SUSPENSE Ultradeep Spectroscopic Program: survey overview and star formation histories of quiescent galaxies at . Astrophys. J. **973**(2), 131 (2024). 10.3847/1538-4357/ad65ff. arXiv:2404.12432 [astro-ph.GA]

[CR179] Smith, M.C., Fielding, D.B., Bryan, G.L., et al.: ARKENSTONE - I. A novel method for robustly capturing high specific energy outflows in cosmological simulations. Mon. Not. R. Astron. Soc. **527**(1), 1216–1243 (2024). 10.1093/mnras/stad3168. arXiv:2301.07116 [astro-ph.GA]

[CR180] Somerville, R.S., Davé, R.: Physical models of galaxy formation in a cosmological framework. Annu. Rev. Astron. Astrophys. **53**, 51–113 (2015). 10.1146/annurev-astro-082812-140951. arXiv:1412.2712 [astro-ph.GA]

[CR181] Somerville, R.S., Hopkins, P.F., Cox, T.J., et al.: A semi-analytic model for the co-evolution of galaxies, black holes and active galactic nuclei. Mon. Not. R. Astron. Soc. **391**, 481–506 (2008). 10.1111/j.1365-2966.2008.13805.x. arXiv:0808.1227

[CR182] Spilker, J.S., Aravena, M., Béthermin, M., et al.: Fast molecular outflow from a dusty star-forming galaxy in the early Universe. Science **361**(6406), 1016–1019 (2018). 10.1126/science.aap8900. arXiv:1809.01676 [astro-ph.GA] 30190403

[CR183] Spilker, J.S., Aravena, M., Phadke, K.A., et al.: Ubiquitous molecular outflows in massive, dusty galaxies. II. Momentum-driven winds powered by star formation in the early Universe. Astrophys. J. **905**(2), 86 (2020). 10.3847/1538-4357/abc4e6. arXiv:2010.12591 [astro-ph.GA]

[CR184] Spitoni, E., Vincenzo, F., Matteucci, F.: New analytical solutions for chemical evolution models: characterizing the population of star-forming and passive galaxies. Astron. Astrophys. **599**, A6 (2017). 10.1051/0004-6361/201629745. arXiv:1605.05603 [astro-ph.GA]

[CR185] Springel, V., Hernquist, L.: Formation of a spiral galaxy in a major merger. Astrophys. J. Lett. **622**, L9–L12 (2005). 10.1086/429486. arXiv:astro-ph/0411379

[CR186] Steidel, C.C., Erb, D.K., Shapley, A.E., et al.: The structure and kinematics of the circumgalactic medium from far-ultraviolet spectra of galaxies. Astrophys. J. **717**, 289–322 (2010). 10.1088/0004-637X/717/1/289. arXiv:1003.0679

[CR187] Steinborn, L.K., Dolag, K., Hirschmann, M., et al.: A refined sub-grid model for black hole accretion and AGN feedback in large cosmological simulations. Mon. Not. R. Astron. Soc. **448**(2), 1504–1525 (2015). 10.1093/mnras/stv072. arXiv:1409.3221 [astro-ph.GA]

[CR188] Stevenson, S.D., Carnall, A.C., Leung, H.H., et al.: PRIMER and JADES reveal an abundance of massive quiescent galaxies at . Mon. Not. R. Astron. Soc. **545**(3), staf2087 (2026). 10.1093/mnras/staf2087. arXiv:2509.06913 [astro-ph.GA]

[CR189] Suess, K.A., Kriek, M., Price, S.H., et al.: Dissecting the size-mass and -mass relations at : galaxy mass profiles and color gradients as a function of spectral shape. Astrophys. J. **915**(2), 87 (2021). 10.3847/1538-4357/abf1e4. arXiv:2101.05820 [astro-ph.GA]

[CR190] Suess, K.A., Beverage, A.G., Kriek, M., et al.: Cold gas in a post-starburst pair in the HeavyMetal Survey: major mergers as a pathway to retain gas in quenched galaxies. Astrophys. J. **993**(2), 158 (2025). 10.3847/1538-4357/ae0933. arXiv:2506.14361 [astro-ph.GA]

[CR191] Sun, Y., Lee, G.H., Zabludoff, A.I., et al.: Evolution of gas flows along the starburst to post-starburst to quiescent galaxy sequence. Mon. Not. R. Astron. Soc. **528**(4), 5783–5803 (2024). 10.1093/mnras/stae366. arXiv:2310.00424 [astro-ph.GA]

[CR192] Sun, Y., Ji, Z., D’Eugenio, F., et al.: A census of Na D-traced neutral ISM and outflows at . arXiv e-prints (2026a). arXiv:2604.18522 [astro-ph.GA]

[CR193] Sun, Y., Ji, Z., Rieke, G.H., et al.: Extreme neutral outflow in a non-active galactic nucleus quiescent galaxy at . Astrophys. J. **997**(2), 140 (2026b). 10.3847/1538-4357/ae2c83. arXiv:2504.14682 [astro-ph.GA]

[CR194] Szpila, J., Davé, R., Rennehan, D., et al.: The nature and evolution of early massive quenched galaxies in the SIMBA-C simulation. Mon. Not. R. Astron. Soc. **537**(2), 1849–1868 (2025). 10.1093/mnras/staf132. arXiv:2402.08729 [astro-ph.GA]

[CR195] Tacchella, S., Dekel, A., Carollo, C.M., et al.: The confinement of star-forming galaxies into a main sequence through episodes of gas compaction, depletion and replenishment. Mon. Not. R. Astron. Soc. **457**(3), 2790–2813 (2016). 10.1093/mnras/stw131. arXiv:1509.02529 [astro-ph.GA]

[CR196] Tacconi, L.J., Genzel, R., Sternberg, A.: The evolution of the star-forming interstellar medium across cosmic time. Annu. Rev. Astron. Astrophys. **58**, 157–203 (2020). 10.1146/annurev-astro-082812-141034. arXiv:2003.06245 [astro-ph.GA]

[CR197] Taylor, E., Maltby, D., Almaini, O., et al.: High-velocity outflows persist up to 1 Gyr after a starburst in recently quenched galaxies at . Mon. Not. R. Astron. Soc. **535**(2), 1684–1692 (2024). 10.1093/mnras/stae2463. arXiv:2411.00102 [astro-ph.GA]

[CR198] Taylor, E., Carnall, A.C., Maltby, D., et al.: The JWST EXCELS survey: outflows in quiescent and recently quenched galaxies are likely relics from episodic AGN activity. Mon. Not. R. Astron. Soc. **549**(3), stag918 (2026). 10.1093/mnras/stag918

[CR199] Toft, S., Smolčić, V., Magnelli, B., et al.: Submillimeter galaxies as progenitors of compact quiescent galaxies. Astrophys. J. **782**(2), 68 (2014). 10.1088/0004-637X/782/2/68. arXiv:1401.1510 [astro-ph.GA]

[CR200] Tremonti, C.A., Moustakas, J., Diamond-Stanic, A.M.: The discovery of 1000 km s^−1^ outflows in massive poststarburst galaxies at . Astrophys. J. Lett. **663**, L77–L80 (2007). 10.1086/520083. arXiv:0706.0527

[CR201] Trussler, J., Maiolino, R., Maraston, C., et al.: Both starvation and outflows drive galaxy quenching. Mon. Not. R. Astron. Soc. **491**(4), 5406–5434 (2020). 10.1093/mnras/stz3286. arXiv:1811.09283 [astro-ph.GA]

[CR202] Valentino, F., Tanaka, M., Davidzon, I., et al.: Quiescent galaxies 1.5 billion years after the Big Bang and their progenitors. Astrophys. J. **889**(2), 93 (2020). 10.3847/1538-4357/ab64dc. arXiv:1909.10540 [astro-ph.GA]

[CR203] Valentino, F., Heintz, K.E., Brammer, G., et al.: Gas outflows in two recently quenched galaxies at and 7. Astron. Astrophys. **699**, A358 (2025). 10.1051/0004-6361/202553908. arXiv:2503.01990 [astro-ph.GA]

[CR204] Valentino, F., Ito, K., Farcy, M., et al.: High-ionization coronal lines trace quasar-like activity in recently quenched galaxies at high redshift. arXiv e-prints (2026). 10.48550/arXiv.2605.30424. arXiv:2605.30424 [astro-ph.GA]

[CR205] Vayner, A., Wright, S.A., Murray, N., et al.: Galactic-scale feedback observed in the 3C 298 quasar host galaxy. Astrophys. J. **851**(2), 126 (2017). 10.3847/1538-4357/aa9c42. arXiv:1709.03510 [astro-ph.GA]

[CR206] Vayner, A., Zakamska, N., Wright, S.A., et al.: Multiphase outflows in high-redshift quasar host galaxies. Astrophys. J. **923**(1), 59 (2021). 10.3847/1538-4357/ac2b9e. arXiv:2110.00019 [astro-ph.GA]

[CR207] Veilleux, S., Maiolino, R., Bolatto, A.D., et al.: Cool outflows in galaxies and their implications. Astron. Astrophys. Rev. **28**(1), 2 (2020). 10.1007/s00159-019-0121-910.48550/arXiv.2002.07765. arXiv:2002.07765 [astro-ph.GA]

[CR208] Vijayan, A., Krumholz, M.R.: When are galactic winds molecular? Mon. Not. R. Astron. Soc. **532**(4), 4132–4140 (2024). 10.1093/mnras/stae1679. arXiv:2404.14608 [astro-ph.GA]

[CR209] Vijayan, A., Kim, C.G., Armillotta, L., et al.: Kinematics and dynamics of multiphase outflows in simulations of the star-forming galactic interstellar medium. Astrophys. J. **894**(1), 12 (2020). 10.3847/1538-4357/ab8474. arXiv:1911.07872 [astro-ph.GA]

[CR210] Vijayan, A., Krumholz, M.R., Wibking, B.D.: QUOKKA-based understanding of outflows (QED) - I. Metal loading, phase structure, and convergence testing for solar neighbourhood conditions. Mon. Not. R. Astron. Soc. **527**(4), 10095–10110 (2024). 10.1093/mnras/stad3816. arXiv:2309.07955 [astro-ph.GA]

[CR211] Wakker, B.P., Mathis, J.S.: Dependence of gas-phase abundances in the interstellar medium on column density. Astrophys. J. Lett. **544**(2), L107–L110 (2000). 10.1086/317316. arXiv:astro-ph/0010045 [astro-ph]

[CR212] Ward, S.R., Costa, T., Harrison, C.M., et al.: AGN-driven outflows in clumpy media: multiphase structure and scaling relations. Mon. Not. R. Astron. Soc. **533**(2), 1733–1755 (2024). 10.1093/mnras/stae1816. arXiv:2407.17593 [astro-ph.GA]

[CR213] Weibel, A., de Graaff, A., Setton, D.J., et al.: RUBIES reveals a massive quiescent galaxy at . Astrophys. J. **983**(1), 11 (2025). 10.3847/1538-4357/adab7a. arXiv:2409.03829 [astro-ph.GA]

[CR214] Weinberger, R., Springel, V., Hernquist, L., et al.: Simulating galaxy formation with black hole driven thermal and kinetic feedback. Mon. Not. R. Astron. Soc. **465**(3), 3291–3308 (2017). 10.1093/mnras/stw2944. arXiv:1607.03486 [astro-ph.GA]

[CR215] Weiner, B.J., Coil, A.L., Prochaska, J.X., et al.: Ubiquitous outflows in DEEP2 spectra of star-forming galaxies at . Astrophys. J. **692**, 187–211 (2009). 10.1088/0004-637X/692/1/187. arXiv:0804.4686

[CR216] Weldon, A., Reddy, N.A., Coil, A.L., et al.: The MOSDEF survey: properties of warm ionized outflows at . Mon. Not. R. Astron. Soc. **531**(4), 4560–4576 (2024). 10.1093/mnras/stae1428. arXiv:2404.05725 [astro-ph.GA]

[CR217] Weng, S., Sadler, E.M., Foster, C., et al.: Observations of cold extragalactic gas clouds at towards PKS 1610-771. Mon. Not. R. Astron. Soc. **512**(3), 3638–3650 (2022). 10.1093/mnras/stac747. arXiv:2109.10875 [astro-ph.GA]

[CR218] Weng, S., Saintonge, A., Pieri, M.M., et al.: Peering down the barrel with DESI DR2: 10 000+ inflows at reveal how galaxies accrete cold gas. arXiv e-prints (2026). 10.48550/arXiv.2605.02999. arXiv:2605.02999 [astro-ph.GA]

[CR219] Whitaker, K.E., Bezanson, R.: Quenching of star formation in massive galaxies. arXiv e-prints (2026). 10.48550/arXiv.2606.12156. arXiv:2606.12156 [astro-ph.GA]

[CR220] Whitaker, K.E., Williams, C.C., Mowla, L., et al.: Quenching of star formation from a lack of inflowing gas to galaxies. Nature **597**(7877), 485–488 (2021). 10.1038/s41586-021-03806-7. arXiv:2109.10384 [astro-ph.GA] 34552255

[CR221] Wild, V., Almaini, O., Cirasuolo, M., et al.: A new method for classifying galaxy SEDs from multiwavelength photometry. Mon. Not. R. Astron. Soc. **440**(2), 1880–1898 (2014). 10.1093/mnras/stu212. arXiv:1401.7878 [astro-ph.CO]

[CR222] Wild, V., Almaini, O., Dunlop, J., et al.: The evolution of post-starburst galaxies from to 0.5. Mon. Not. R. Astron. Soc. **463**(1), 832–844 (2016). 10.1093/mnras/stw1996. arXiv:1608.00588 [astro-ph.GA]

[CR223] Wild, V., Taj Aldeen, L., Carnall, A., et al.: The star formation histories of post-starburst galaxies. Mon. Not. R. Astron. Soc. **494**(1), 529–548 (2020). 10.1093/mnras/staa674. arXiv:2001.09154 [astro-ph.GA]

[CR224] Williams, C.C., Spilker, J.S., Whitaker, K.E., et al.: ALMA measures rapidly depleted molecular gas reservoirs in massive quiescent galaxies at . Astrophys. J. **908**(1), 54 (2021). 10.3847/1538-4357/abcbf6. arXiv:2012.01433 [astro-ph.GA]

[CR225] Wolfire, M.G., Hollenbach, D., McKee, C.F., et al.: The neutral atomic phases of the interstellar medium. Astrophys. J. **443**, 152 (1995). 10.1086/175510

[CR226] Wright, R.J., Somerville, R.S., CdP, L., et al.: The baryon cycle in modern cosmological hydrodynamical simulations. Mon. Not. R. Astron. Soc. **532**(3), 3417–3440 (2024). 10.1093/mnras/stae1688. arXiv:2402.08408 [astro-ph.GA]

[CR227] Wu, P.F.: Ejective feedback as a quenching mechanism in the first 1.5 billion years of the universe: detection of neutral gas outflow in a recently quenched galaxy. Astrophys. J. **978**(2), 131 (2025). 10.3847/1538-4357/ad98ef. arXiv:2409.00471 [astro-ph.GA]

[CR228] Wu, P.F., van der Wel, A., Bezanson, R., et al.: Fast and slow paths to quiescence: ages and sizes of 400 quiescent galaxies from the LEGA-C Survey. Astrophys. J. **868**(1), 37 (2018). 10.3847/1538-4357/aae822. arXiv:1809.01211 [astro-ph.GA]

[CR229] Xu, Y., Ouchi, M., Nakajima, K., et al.: Astrophys. J. **984**(2), 182 (2025). 10.3847/1538-4357/adc733

[CR230] Zabludoff, A.I., Zaritsky, D., Lin, H., et al.: The environment of “E + A” galaxies. Astrophys. J. **466**, 104 (1996). 10.1086/177495. arXiv:astro-ph/9512058

[CR231] Zakamska, N.L., Hamann, F., Pâris, I., et al.: Discovery of extreme [O III] 5007 Å outflows in high-redshift red quasars. Mon. Not. R. Astron. Soc. **459**(3), 3144–3160 (2016). 10.1093/mnras/stw718. arXiv:1512.02642 [astro-ph.GA]

[CR232] Zanella, A., Valentino, F., Gallazzi, A., et al.: The large molecular gas fraction of post-starburst galaxies at . Mon. Not. R. Astron. Soc. **524**(1), 923–939 (2023). 10.1093/mnras/stad1821. arXiv:2306.08120 [astro-ph.GA]

[CR233] Zhong, Y., Inoue, A.K., Sugahara, Y., et al.: Revisiting the Dragonfly galaxy - I. High-resolution ALMA and VLA observations of the radio hotspots in a hyper-luminous infrared galaxy at . Mon. Not. R. Astron. Soc. **522**(4), 6123–6136 (2023). 10.1093/mnras/stad1428. arXiv:2305.03979 [astro-ph.GA]

[CR234] Zhu, P., Ito, K., Valentino, F., et al.: There and back again? Neutral outflows in quiescent galaxies. arXiv e-prints (2026). 10.48550/arXiv.2602.17767. arXiv:2602.17767 [astro-ph.GA]

[CR235] Zinger, E., Pillepich, A., Nelson, D., et al.: Ejective and preventative: the IllustrisTNG black hole feedback and its effects on the thermodynamics of the gas within and around galaxies. Mon. Not. R. Astron. Soc. **499**(1), 768–792 (2020). 10.1093/mnras/staa2607. arXiv:2004.06132 [astro-ph.GA]

[CR236] Zolotov, A., Dekel, A., Mandelker, N., et al.: Compaction and quenching of high-z galaxies in cosmological simulations: blue and red nuggets. Mon. Not. R. Astron. Soc. **450**(3), 2327–2353 (2015). 10.1093/mnras/stv740. arXiv:1412.4783 [astro-ph.GA]

[CR237] Zubovas, K., Maskeliūnas, G.: Life after AGN switch off: evolution and properties of fossil galactic outflows. Mon. Not. R. Astron. Soc. **524**(4), 4819–4840 (2023). 10.1093/mnras/stad1661. arXiv:2306.00518 [astro-ph.GA]

